# Investigation of Hyperfine Interactions in Molecular Spin Qubits Constructed from A Nitronyl-Nitroxide Ligand and Transition Metal Ions

**DOI:** 10.1021/acs.inorgchem.5c05585

**Published:** 2026-03-13

**Authors:** Daniel O. T. A. Martins, Cristian A. Spinu, Alena Sheveleva, Mihaela Hillebrand, Floriana Tuna, Marius Andruh

**Affiliations:** † Department of Chemistry and Photon Science Institute, 5292 University of Manchester, Oxford Road, Manchester M13 9PL, U.K.; ‡ Faculty of Chemistry, University of Bucharest, Regina Elisabeta Blvd. 4-12, Bucharest 030018, Romania; § C. D. Nenitzescu Institute of Organic and Supramolecular Chemistry of the Romanian Academy, Splaiul Independentei, 202B, Bucharest 060023, Romania

## Abstract

The qubit behavior of two *S* = 1/2 heterospin complexes with the general formula (Et_3_NH)­[M­(hfac)_2_L] has been investigated by pulse EPR methods (M = Zn (**1**) and Ni (**2**), hfac^*–*^ is the coligand hexafluoroacetylacetonate and L^*–*^ is the deprotonated nitrophenol-substituted NIT radical 2-(2-hydroxy-3-methoxy-5-nitrophenyl)-4,4,5,5-tetramethyl-4,5-dihydro-1*H*-imidazol-3-oxide-1-oxyl). Robust quantum coherence is observed in both compounds. At 100 K, **1** shows a longer phase memory time, *T*_m_ (0.9 μs) than **2 (**0.12 μs), while at very low temperatures, the opposite is true (**1:** 1.78 μs at 5.2 K; **2**: 3.7 μs at 5.5 K). With CPMG detection, longer *T*_m_ up to18 μs (**1**) and 7 μs (**2**) at 5 K is measured. The spin–lattice relaxation time (*T*_1_) is also longer for **1** than for **2**, due to strong spin-orbit coupling in the latter. HYSCORE and ENDOR investigations quantified the hyperfine couplings to ^19^F, ^1^H, ^67^Zn, and ^14^N, providing clear insights into the low temperature decoherence paths in the two qubits.

## Graphical abstract

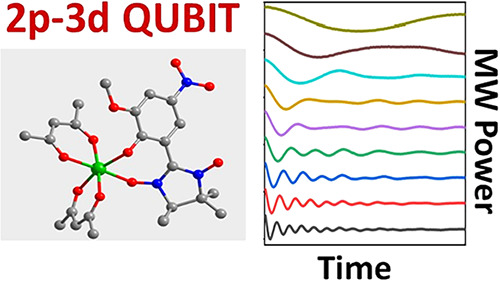

## Introduction

The interest in nitronyl-nitroxide (NIT) radicals in molecular magnetism dates back to the 1980s with the pioneering work by Gatteschi and collaborators.^1^ The compounds are paramagnetic, with an unpaired electron equally delocalized between two NO groups (Scheme 1). Functionalization of NIT radicals with coordinating R groups can be readily achieved,^2^ allowing to access 2p–3d heterospin complexes with interesting structures and magnetic properties.^3,4^

**Scheme 1:**
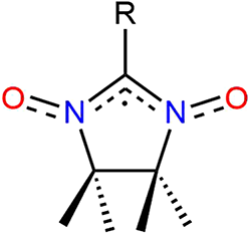
Schematic Representation of the Nitronyl-Nitroxide Radicals

NIT radicals have long relaxation times,^5^ and thus we were interested to explore their potential for quantum information processing (QIP). The central concept of QIP is that the information is encoded in a two-level quantum register, the *qubit* (or quantum bit), which can generate and control a large number of states that are other than the common 0 and 1 used in classical computation, but a superposition of those.^6^ This “quantum parallelism” greatly improves the processing speeds, theoretically enabling the quantum computer to solve some problems currently impossible to achieve with classical analogues. There are several candidates for the physical implementation of qubits, such as quantum dots,^7^ nitrogen vacancies in diamond^8^ and superconducting circuits,^9^ to name a few, and significant progress has been made over the years.^10^ Among the most prominent alternatives, magnetic molecules offer unique advantages not seen in other archetypes.^11^ In these systems, the electronic spin can be understood as the qubit, and they benefit from the inherent homogeneity of molecular systems and great chemical versatility from the possibility to tune the ligands and thus the magnetic behavior.^6^

An important condition to be fulfilled by qubits is a prolonged coherence time that assures the respective qubits retain their properties during calculations.^10^ However, this coherence can be lost to interactions of the qubit with its environment. For molecular systems, quantum decoherence usually occurs via the interaction of the electronic spin with the nuclear spins from its vicinity (hyperfine interactions),^6,12^ lattice vibrations (phonons),^13a^ and interactions of the qubit with neighboring electron spins (dipolar interactions).^13^ Studies by one of us proved that the electronic structure of the qubit is also important, with prolonged relaxation times being observed for systems with reduced orbital angular momentum in the ground state.^14^ All of these factors must be taken into account in the design of qubit candidates. Among *S* = 1/2 qubits, very good qubit performance was achieved with vanadium­(IV) complexes incorporating nuclear spin-free ligands;^15^ chelating ligands;^16a^ or π-conjugated macrocycles.^16b^ Remarkable qubit properties were also reported for copper­(II) porphyrins,^17^ Cu^2+^ doped metal organic frameworks,^18^ Cu­(dtp)^2–^,^19a^ and Ni­(dtp)_2_^–^ (mnt = maleonitriledithiolato).^19b,19c^ An organometallic Y^2+^ complex studied by one of us demonstrated prolonged coherence and Rabi oscillations at room temperature in a single crystal.^14a^ Sessoli et al. also observed Rabi oscillations at room temperature in a vanadyl phthalocyanine complex,^16b^ while Freedman et al.^20^ and van Slageren et al.^19a^ achieved room temperature quantum coherence with square-planar copper­(II) complexes. *S* = 1 qubits, e.g., chromium­(IV), molybdenum­(IV), vanadium­(III), have been characterized recently.^21^

Several 4f complexes,^13c,14b,22,23^ and nitronyl-nitroxide molecules,^24^ separately, have been tested as spin qubits. In a recent study, we have shown that heterospin qubit systems can be achieved as well.^25^ Perchlorotriphenylmethyl radicals also demonstrate very good qubit performances.^26^

Herein, we evaluate the qubit properties of two metallo-nitronyl-nitroxide complexes of general formula (Et_3_NH)­[M­(hfac)_2_L], where M = Zn (**1**) or Ni (**2**), hfac^*–*^ is the coligand hexafluoroacetylacetonate, and L^–^ is the deprotonated nitrophenol-substituted NIT radical 2-(2-hydroxy-3-methoxy-5-nitrophenyl)-4,4,5,5-tetramethyl-4,5-dihydro-1*H*-imidazol-3-oxide-1-oxyl (Scheme 2). The crystal structures and magnetic properties of the two compounds were reported elsewhere.^27^

**Scheme 2:**
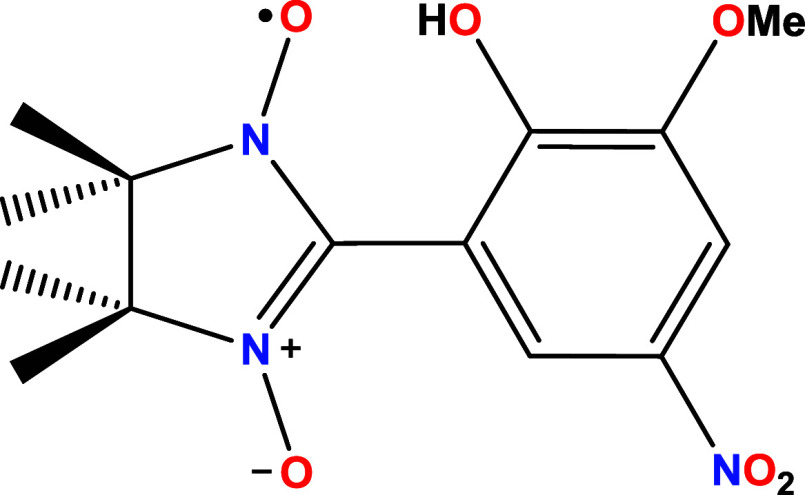
Schematic Representation of the Nitronyl-Nitroxide Radical (HL) Used in This Work

These compounds are isostructural and characterized by an *S* = 1/2 ground state. They are very stable since all three ligands are chelated to the metal ion. The key difference is in spin density, which is primarily located in the NIT fragment for **1**, and on nickel in **2**, based on DFT studies.^27^ The *S* = 1/2 ground state of **2** arises from strong antiferromagnetic exchange (*J*_NiRad_ = −351 cm^–1^) between the radical and the nickel­(II) ion, and is well isolated below 120 K.^27^ These characteristics make the compounds ideal models to examine how metals and ligands affect the qubit properties. Here we employ pulse EPR hyperfine methods to evaluate electron spin - nuclear spin interactions that can contribute to decoherence. Spin densities at ligand atoms correlates with the magnitude of hyperfine couplings.^14b^ Hfac^–^ ligands were deliberately chosen for this study as they contain ^19^F nuclei that are visible in HYSCORE and ENDOR. Their different Larmor frequencies eliminate uncertainties arising from solvents whose protons can also contribute to decoherence. Moreover, the rigidity of the six-membered chelates could increase the energy of the vibrational modes,^28^ further favoring long phase memory times. Further validation of spin density transfer is obtained from the interactions of the qubit with ^1^H, ^14^N, and ^67^Zn nuclei, which are also examined.

## Results and Discussion

Both compounds are isostructural, and we recall here the structure of the zinc derivative (Figure 1). In both compounds, the metal ions show an octahedral geometry, being coordinated by the three chelating ligands (two hfac^–^ and one deprotonated radical).

**Figure 1:**
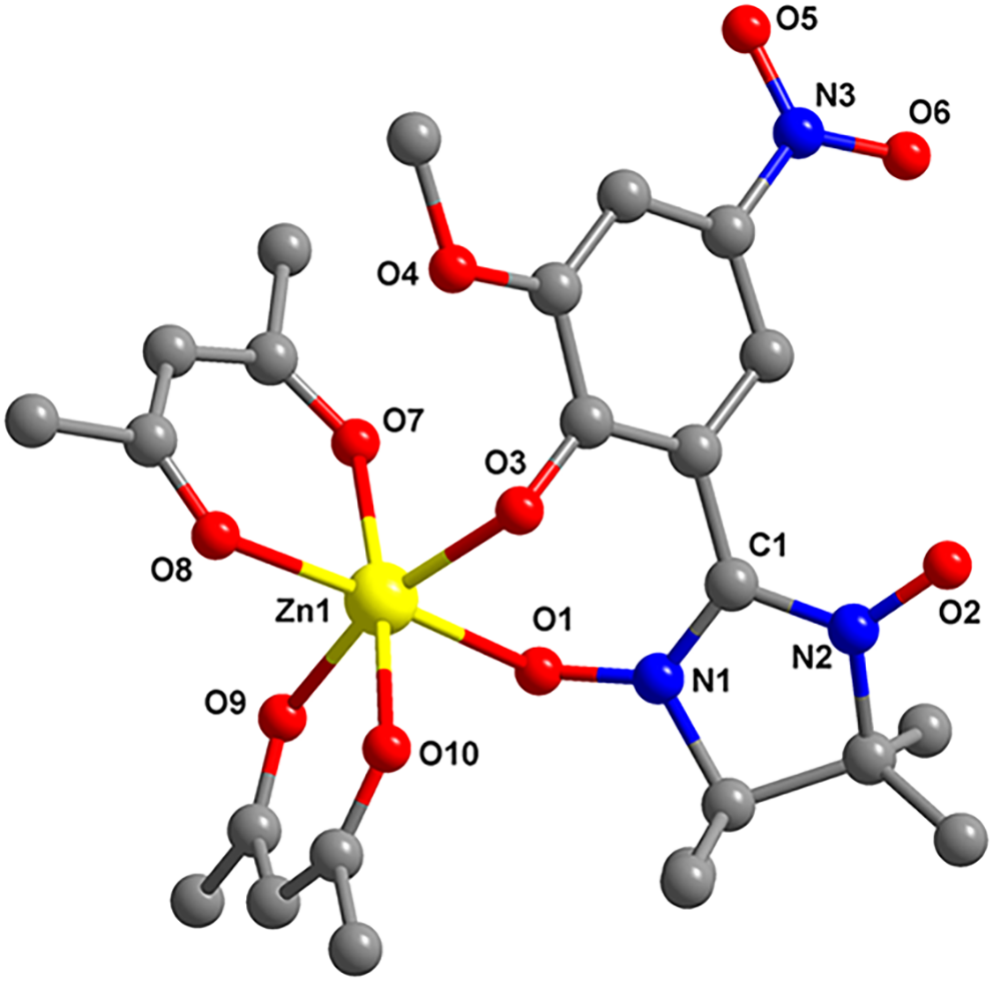
Crystal structure of the [Zn­(hfac)_2_L]^−^ complex anion in complex **1**.

### Continuous-Wave (CW) EPR

The CW-EPR spectra of **1** are consistent with an *S* = 1/2 spin state due to the nitronyl-nitroxide radical (ESI Figures S1 and S2), since zinc­(II) has a 3d^10^ electronic configuration and thus is diamagnetic. Data were simulated using the spin Hamiltonian (1) that takes into account the electron Zeeman interaction (EZI) and the hyperfine interaction (HFI)

1 $$Ĥ=\mu_{B}BgŜ+\sum_{i=N_{1}\;,N_{2}\;}ŜA_{i}{Î}_{i}$$

where *S* = 1/2, *I*_N_ = 1 is the nuclear spin of ^14^N (99.6% natural abundance), *A*_*N*1,*N*2_ are the hyperfine tensors of the two equivalent nitrogen nuclei present in the nitronyl-nitroxide radical, μ_B_ is the Bohr magneton, and *g* is the molecular *g*-tensor. Best simulation of the spectra, using EasySpin 6.0,^29^ was achieved with the parameters in Table 1. The *g*- and *A*-anisotropy parameters may be obtained from the immobilized sample experiments (i.e., powder and frozen solution), the fluid solution spectra allow the determination of the isotropic values, with the parameters defined as *g*_iso_ = (*g*_*x*_ + *g*_*y*_ + *g*_*z*_)/3 and *A*_iso_ = (*A*_*x*_ + *A*_*y*_ + *A*_*z*_)/3, provided that they are interpreted in units of frequency, not energy or magnetic field. Only the biggest component of the hyperfine tensor (*A*_*x*_) could be determined from CW experiments, and *A*_*y*,*z*_ was determined only from pulse EPR techniques.

**Table 1:** Extracted EPR Parameters for **1** and **2** Were from Simulations^a^

	conditions	*g*-values	*A* ^N1,2^ (MHz)
**1**	frozen solution and powder	*g*_*x*_ = 2.0103(5)	*A*_*x*_ = 54(2)
		*g*_*y*_ = 2.0060(5)	*A*_*y*_ = 5.2
		*g*_*x*_ = 2.0019(5)	*A*_*z*_ = 4.4
	fluid solution	*g*_iso_ = 2.0060(5)	*A*_iso_ = 21.2
**2**	frozen solution and powder	*g*_*x*_ = 2.32(1)	N/A
		*g*_*y*_ = 2.28(1)	
		*g*_*x*_ = 2.26(1)	

a The error bars are a result of simultaneous simulations of the different experimental conditions.

The spectra of **2** are also consistent with a *S* = 1/2 spin state (ESI Figures S3 and S4) that is stabilized by antiferromagnetic coupling between nickel­(II) (*S =* 1) and the nitronyl-nitroxide radical (*S =* 1/2).^27^ Its EPR signal is rhombic, providing *g* = 2.32, 2.28, and 2.26. These values suggest a stronger spin orbit coupling (SOC) in **2** compared to **1**, with most of the electron density residing on nickel, in full agreement with the results of density functional theory (DFT) (Table S9).^27^ We note that the *g*-anisotropy is more prominent in the frozen solution than powder spectra due to dipolar interactions in the solid state, causing some broadening of the EPR lines. Nevertheless, all spectra of **2** were nicely modeled with a spin Hamiltonian that only accounts for the EZI contribution (Figures S3 and S4).

Variable temperature EPR experiments on **1** and **2** show that the compounds retain their spectral features over a wide temperature range (Figure S5), encouraging a detailed pulse EPR investigation.

### Echo-Detected Field-Sweep (EDFS) Spectra

Spin echoes were generated with a standard Hahn-echo sequence for frozen solutions of compounds **1** and **2**. The obtained X-band EDFS spectra (Figure 2) match the absorption curves of their continuous-wave counterparts (ESI Figures S1 and S3) and are simulated with the same spin Hamiltonian parameters (Table 1).

**Figure 2:**
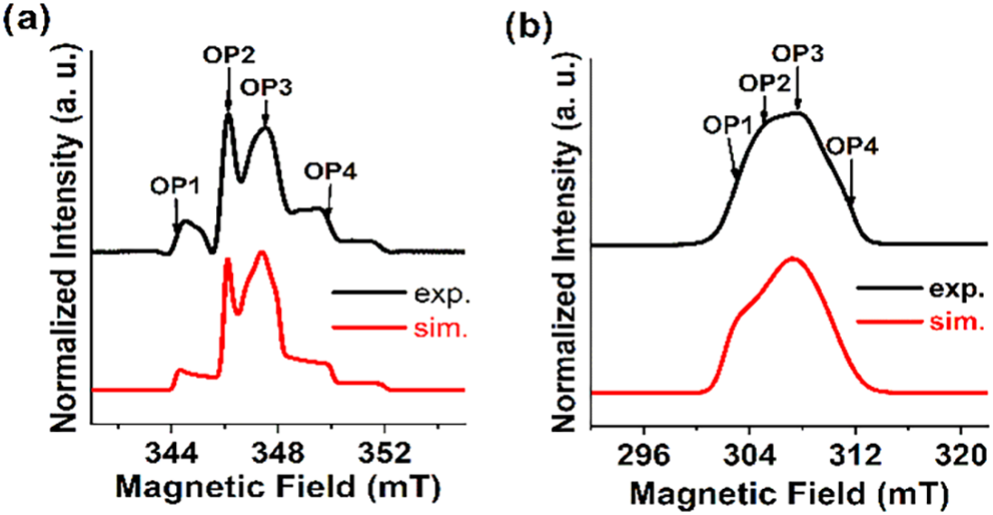
Experimental (black) and simulated (red) X-band (ca. 9.70 GHz) EDFS spectra of **1** (a) and **2** (b). The arrows mark the observer positions at which time-dependent experiments were performed. Simulation parameters are provided in Table 1.

### Relaxation Times

Relaxation times were measured at different observer positions (OPs) as indicated in Figure 2, and at temperatures from 5 to 100 K (Figures 3 and S6–S8, and Tables S1–S4). The spin–lattice relaxation time, *T*_1_, for **1** is very long, reaching 4.1 ms at 5.2 K, and is still as long as 197 μs at 100 K at OP1 (Figures 3, S6, and Table S1). Compared to free nitronyl-nitroxide organic radicals,^24^ compound **1** shows faster spin–lattice relaxation, which is unsurprising considering the large number of nuclear spins (^1^H, ^19^F, ^67^Zn, ^14^N) in the molecule that can engage in hyperfine interactions with the electron spin.^12,14^ The rotation of the CF_3_ groups in particular can cause relaxation through a mechanism known as spectral diffusion (SD).^12^ Fitting of *T*_1_ data included a temperature-dependent *T*_SD_ term to account for spectral diffusion effects.^6a^ Obtained *T*_SD_ values are significantly smaller than *T*_1_ and follow the same temperature trend, i.e., they increase with the decreasing of the temperature. Compound **2** shows a shorter *T*_1_ of 0.9 ms at 5.5 K, decreasing to 0.3 μs at 100 K (Figures 3, S7, and Table S3). *T*_SD_ is also smaller for **2** than for **1** at comparable temperatures. The stronger temperature dependence of *T*_1_ for **2** is likely caused by unquenched orbital angular momentum associated with the nickel­(II) ion.^14a,20^

**Figure 3:**
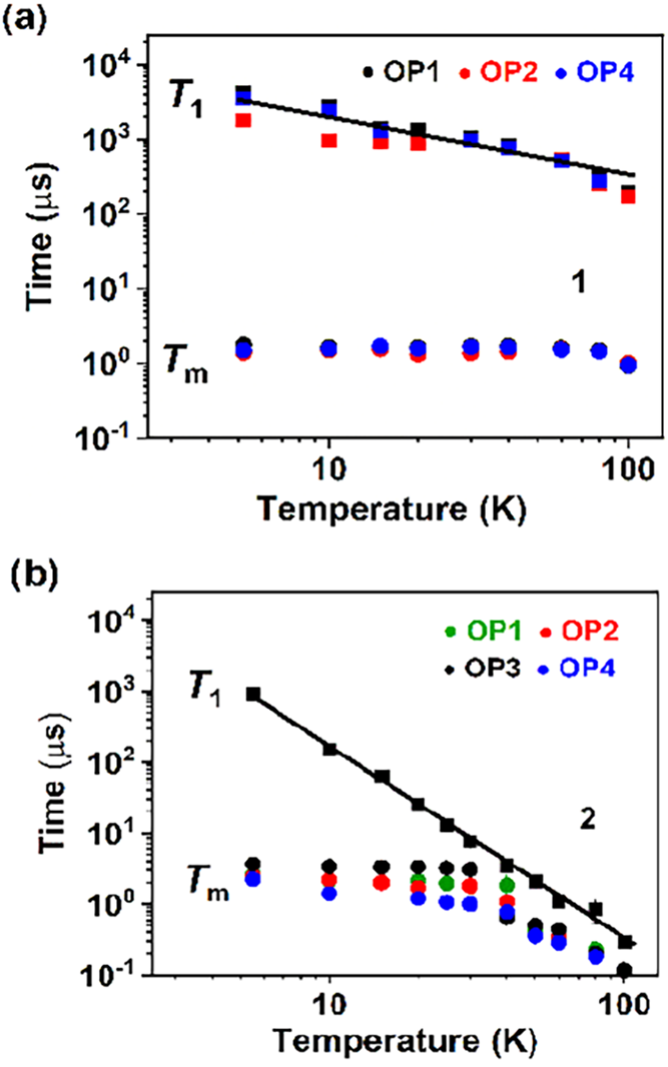
Temperature dependence of the relaxation times *T*_1_ and *T*_m_ of compounds **1** (a) and **2** (b). The black line is a simultaneous fit of all *T*_1_ data with *T*_1_ = (*aT* + *bT^n^*)^−1^.

The phase memory time, *T*_m_, for **1** is almost temperature-independent (ca. 1.50 μs) below 80 K and close to 1 μs at 100 K (Figures 3, S6, and Table S2). The longest memory time was measured at OP_1_ (1.78(5) μs at 5.2 K). For **2**, *T*_m_ is temperature-independent only below 30 K at approximately 2 μs at OP1 and OP2, 3.0 μs at OP3, and 1.0 μs at OP4; it reduces drastically as the temperature is increased (Figures 3, S8, and Table S4). The longest memory time for **2** is measured at OP_3_, varying from 3.7(1) μs at 5.5 K to 0.12 μs at 100 K (Figures 3, S8b, and Tables S4). Thus, at elevated temperature, *T*_m_ of **1** is superior to that of **2**, which is as expected, considering the faster spin–lattice relaxation in the latter. The spin–lattice relaxation time acts as an upper limit to *T*_2_, as it is often observed that *T*_2_ ≤ 2*T*_1_.^6^ However, at low temperature, the nickel compound **2** has a much greater *T*_m_ than **1**. The reason for such a behavior is not immediately clear. One possible reason is a stronger decoherence in **1** due to its electronic density residing in the proximity of the ^14^N nuclei of the NIT fragment. However, compound **2** is also expected to be affected by decoherence due to many ^19^F nuclei present in the molecule.

Decoherence from the ESEEM effect can be partially overcome by using a pulse sequence that decouples the electron spin from the environment;^14b^ thus, transverse relaxation times were also determined using Carr–Purcell–Meiboom–Gill (CPMG) sequence.^30^ *T*_2_^CPMG^ as high as 18 μs was observed for **1** at OP4 and of 7 μs for **2** at OP3 and 5.5 K (ESI Figure S9, Tables S5, and S6). Compared to *T*_m_, there is a 10-fold increase in the relaxation time of **1** and 2-fold for **2**. We expected CPMG time constants longer than phase memory because of the great number of spin-active nuclei in the radical moiety and hfac groups.^30^ Thus, reduction of ESEEM effects using the CPMG approach inverses the coherence time trend, with a longer *T*_2_^CPMG^ being observed for **1** than for **2**. This clearly indicates that hyperfine couplings play a crucial role in qubit decoherence at low temperatures.

### Rabi Oscillations

Transient nutation experiments were performed to assess whether the spin state could be coherently manipulated and placed in arbitrary superposition states within the Bloch sphere. Rabi oscillations were detected for **1** and **2** at different magnetic field positions under different applied microwave power (Figures 4, S10, and S11). They decay in a shorter time than the observed *T*_m_ due to additional dephasing induced by the microwave field used to drive the system through superposition states, with the stronger and more inhomogeneous microwave fields expected to cause stronger Rabi oscillation decay.^6c,14^ In the context of quantum computation, the time period between a maximum and an adjacent minimum corresponds to the time needed to execute a single-qubit logic operation.^15c,31^ Crucially, the qubit operation time parameter needs to be notably smaller than the coherence duration (i.e., *T*_2_ time) in order the qubit to be functional. Interestingly, this operation time is almost identical for compounds **1** and **2**, despite having very different *g* factors. We measured 71 and 72 ns for **1** and **2**, respectively, from nutation data at 16 dB attenuation, decreasing to 12 ns at 1 dB (Figure S12). Thus, the operation time is shortened drastically for higher power pulses.

**Figure 4:**
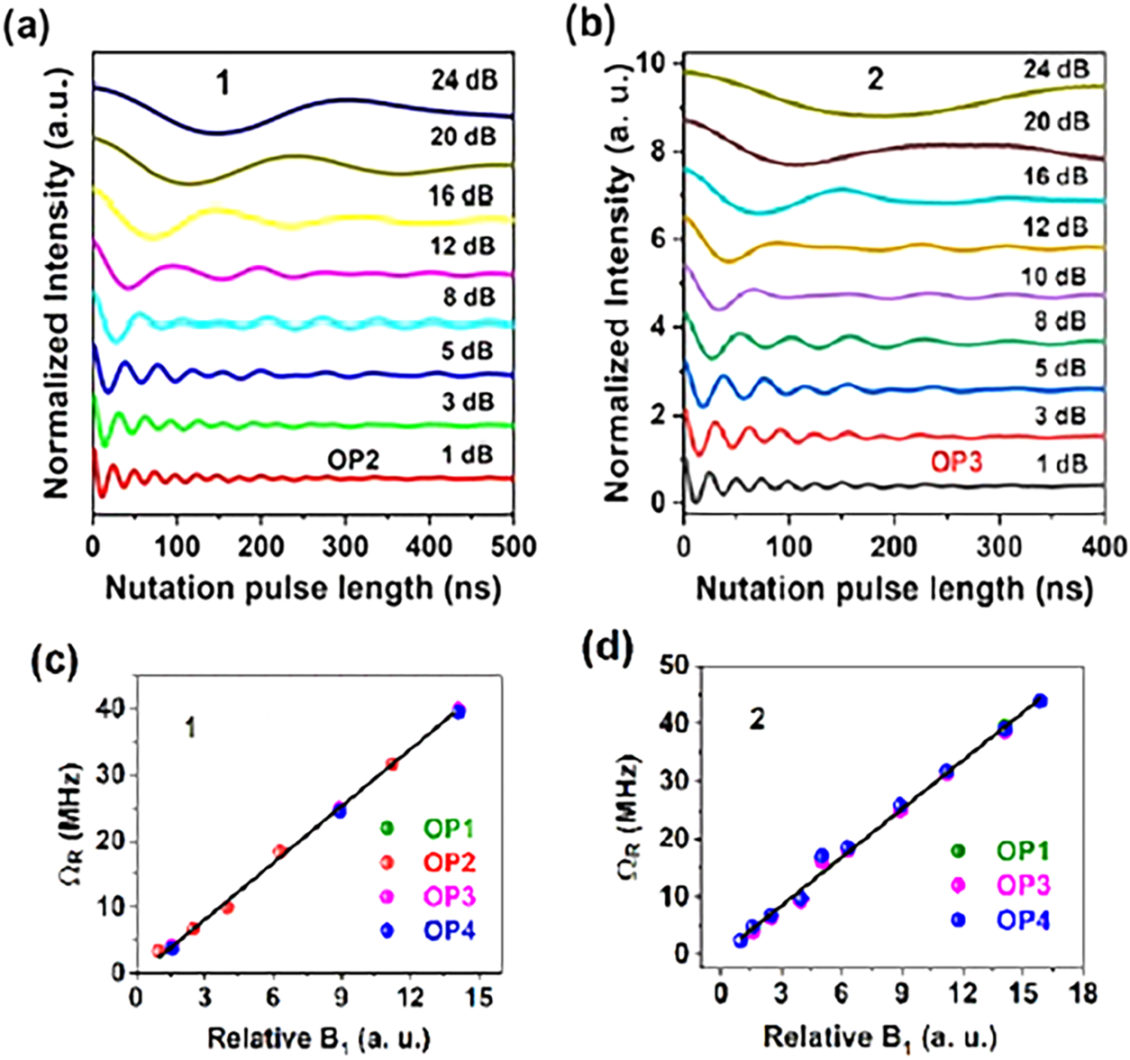
Rabi oscillations acquired with different microwave attenuations for (a) **1** and (b) **2**. (c, d) *B*_1_ dependence of the Rabi frequency, Ω_R_, at selected observer positions. The straight line is a guide to the eye, emphasizing the linear dependence.

The frequency of these oscillations, the so-called Rabi frequency, Ω_R_, is proportional to *g*μ_*B*_ *B*_1_/*ℏ*, thus varies linearly with the magnetic field of the microwave radiation, *B*_1_. Such a linear dependence is observed for all observable positions of **1** and **2** (Figure 4c,d). Observation of coherent Rabi oscillations establishes **1** and **2** as viable qubit candidates. The number of spin-flips a qubit can perform within the *T*_m_ time scale is estimated by a figure of merit, *Q*_M_, defined by 2*T*_m_Ω_R_;^23a^ higher *Q*_M_ assures faster calculations. The *Q*_M_ value for **1** and **2** is 134 and 271, respectively, at 10 K, and is measurable up to 100 K. These values are superior to many reported molecular spin qubits.^14,23^ With *T*_2_^CPMG^ = 18 μs (**1**) and 7 μs (**2**) at 5 K, we predict *Q*_M_ = 1440 and 615, respectively.

### HYSCORE

Electron–nuclear spin–spin interactions are known sources of spin decoherence in molecular spin qubits.^6^ We used HYSCORE (hyperfine sublevel correlation) spectroscopy, a 2D ESEEM (electron spin–echo envelope modulation) technique that correlates nuclear frequencies in the α and β manifolds, to evaluate the hyperfine couplings between the qubit and its surrounding remote spin-active nuclei. The spectra of **1** and **2** are dominated by signals of ^1^H, ^14^N, and ^19^F. In the case of **1**, additional ^67^Zn hyperfine signals are present. Experimental HYSCORE data were simulated according to the Hamiltonian in eq 2, which considers the electron and nuclear Zeeman interactions (NZI), hyperfine interaction, and nuclear quadrupole interaction (NQI).

2 $$Ĥ=\mu_{B}BgŜ-\sum_{i}\mu_{n}g_{\mathrm{ni}}B{Î}_{i}+\sum_{i}ŜA_{i}{Î}_{i}+\sum_{j}{Î}_{j}P_{j}{Î}_{j}$$

where the subscript *i* stands for the spin-active nuclei and *j* for the quadrupole (*I* > 1/2) nuclei. *I*_H_ = *I*_F_ = 1/2 are the nuclear spin of ^1^H (99.9% natural abundance) and ^19^F (100% natural abundance) respectively, *I_N_* = 1 is the nuclear spin of ^14^N (99.6% natural abundance), *I_Zn_* = 5/2 is the nuclear spin of ^67^Zn (4.1% natural abundance), μ_*n*_ is the nuclear magneton, *g_n_* is the nuclear *g*-value, *P* is the NQI tensor described in [Disp-formula eq3],

3 $$P_{j}=\left(\begin{array}{lll} P_{1} & 0 & 0\\ 0 & P_{2} & 0\\ 0 & 0 & P_{3} \end{array}\right)=\frac{e^{2}Q_{j}q_{j}/h}{4I_{j}\left(2I_{j}-1\right)}=\left(\begin{array}{lll} -\left(1-\eta \right) & 0 & 0\\ 0 & -\left(1+\eta \right) & 0\\ 0 & 0 & 2 \end{array}\right)$$

where *Q*_*N*_ = +0.02044(3) *b* is the scalar quadrupole moment of ^14^N and *Q*_*Zn*_ = +0.150(15) *b* of ^67^Zn,^32^ *q* is the second derivative of the electrostatic potential at the nucleus due to all molecular charges outside the nucleus, *e* is the elementary charge, η is the asymmetry parameter, and the other symbols have their usual meaning. The quantity *e* ^2^ *Qq/h* is the quadrupole coupling constant in MHz and arises from two contributions: *eQ* and *eq* from nuclear quadrupole moment and electric field gradient, respectively.^33,34^

^1^H and ^19^F hyperfine signals were observed in the weakly coupled (+,+) quadrant, when |*A*| < 2 ν, in compounds **1** and **2**, centered at the Larmor frequency (ν) and spread by the hyperfine matrix (*A*) along the diagonal (Figure 5). The spectra were simulated considering that the hyperfine matrix has dipolar and isotropic contributions,

4 $$A=\left[\begin{array}{lll} A_{\mathrm{dip}\left(\mathrm{xx}\right)} & A_{\mathrm{dip}\left(\mathrm{xy}\right)} & A_{\mathrm{dip}\left(\mathrm{xz}\right)}\\ A_{\mathrm{dip}\left(\mathrm{yx}\right)} & A_{\mathrm{dip}\left(\mathrm{yy}\right)} & A_{\mathrm{dip}\left(\mathrm{yz}\right)}\\ A_{\mathrm{dip}\left(\mathrm{zx}\right)} & A_{\mathrm{dip}\left(\mathrm{zy}\right)} & A_{\mathrm{dip}\left(\mathrm{zz}\right)} \end{array}\right]+\left[\begin{array}{lll} A_{\mathrm{iso}} & 0 & 0\\ 0 & A_{\mathrm{iso}} & 0\\ 0 & 0 & A_{\mathrm{iso}} \end{array}\right]$$

**Figure 5:**
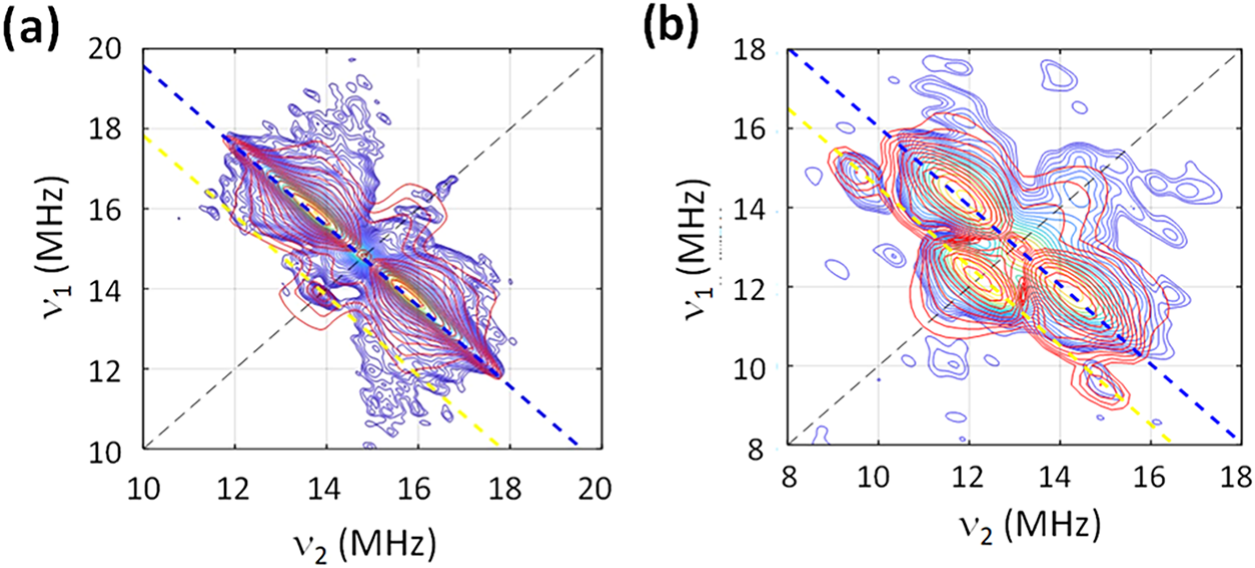
X-band (ca. 9.7 GHz) experimental (blue) and simulation (red) ^1^H, ^19^F-HYSCORE spectra of compound **1** at OP2 and τ = 200 ns (a) and compound **2** at OP2 and τ = 300 ns (b). The black dashed lines mark the diagonal where ν_1_ = ν_2_. The blue and yellow dashed lines mark the Larmor frequencies of ^1^H and ^19^F, respectively.

The dipolar couplings were obtained using the point-dipole model (ESI Tables S7 and S8). They were calculated for each spin-active nuclei independently and averaged for those with indistinguishable crystallographic positions (i.e., CH_3_ and CF_3_ groups). ^19^F spectra of **1** were nicely simulated with the dipolar model alone, considering four fluorine environments (F31,32,33; F35,36,37; F38,39,40; and F42,43,44 in Figure 6), associated with the hfac^*–*^ ligands. Simulation of the corresponding ^1^H spectra considered five proton environments originating from four nitronyl-nitroxide methyl groups (H52,53,54; H55,56,57; H62,63,64; and H65,66,67) and the hydrogens in the aromatic ring at the *ortho* position (H45) with respect to the radical moiety. Consideration of through-space interactions alone did not produce a satisfactory result, but addition of an isotropic component of *A*_iso_ = −0.3 and 2.0(2) MHz for aliphatic and aromatic protons, respectively, led to excellent simulation. Notably, the same spin Hamiltonian reproduces the spectra measured at several other observer positions and for different interpulse delays (ESI Figure S13). The results are in line with previous findings for nitronyl-nitroxide radicals that protons in *ortho* position have larger hyperfines than the methyl hydrogens.^24a^

**Figure 6:**
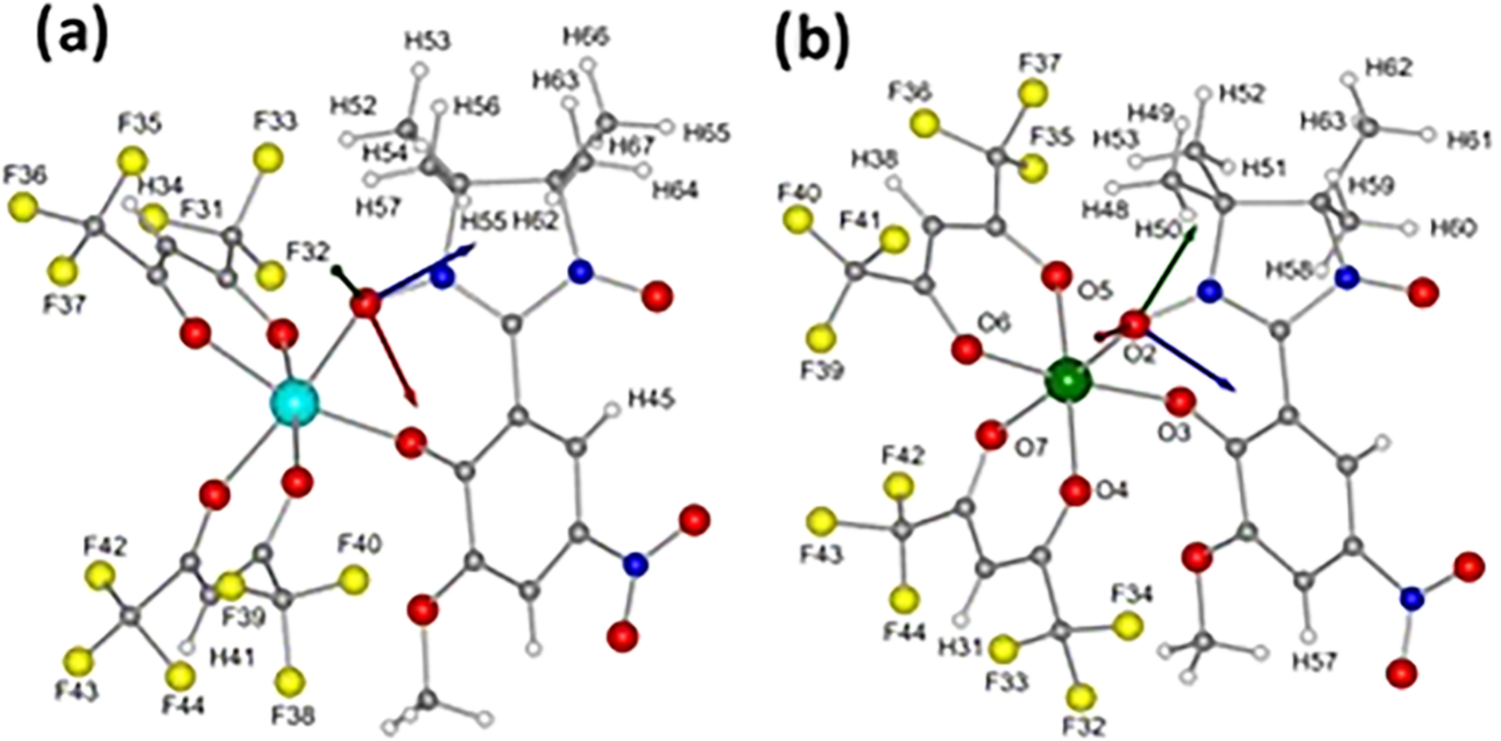
Crystal structures of compounds **1** (a) and **2** (b) overlaid with the molecular *g*-frame (blue: *x*-axis, green: *y*-axis, and red: *z*-axis). Color codes: C, gray; H, white; N, blue; O, red; F, yellow; Zn, teal, and Ni, green. Disorder effects and solvent molecules are not shown for the sake of clarity.

In compound **2**, four fluorine environments were also considered: F32,33,34; F35,36,37; F39,40,41; and F42,43,44 from the *hfac* ^–^ ligands (Figure 6). In this case, the consideration of dipolar interactions alone did not reproduce the experiment. Isotropic hyperfine contribution of *A*_iso_ = −0.5, 2.0, 4.5, and 2.0 MHz, respectively, were added to model the spectra, accounting for the fluorine atoms (ESI Figure S14). The largest isotropic component is assigned to the CF_3_ group closest to the shortest Ni–O bond (Ni–O6, 2.008 Å), and the smallest *A*_iso_ to the fluorine near the longest bond (Ni–O4, 2.052 Å). The isotropic ^19^F hyperfine implies through bond interactions, which are possibly mediated by spin polarization considering the hfac^–^ ligands are chelated to the nickel ion. Six proton environments were considered for the modeling of ^1^H HYSCORE data: four from the radical methyl groups (H48,49,50; H51,52,53; H58,59,60; and H61,62,63 in Figure 6) and two from hfac^*–*^ ligand (H31 and H38 in Figure 6). Isotropic components of *A*_iso_ = 0.6 MHz counting for the methyl protons, and 2.0 MHz for the hfac^*–*^ were used; the same spin Hamiltonian faithfully reproduces the other observer positions and τ-values (ESI Figure S14). Because the hyperfine coupling is proportional to the spin density, these values show that, when compared to **1**, the spin density in **2** is more evenly delocalized over the whole molecule, not only at the nitronyl-nitroxide moiety, because of the presence of a paramagnetic metal ion, nickel­(II). Indeed, the results of DFT calculations indicate larger Mulliken atomic spin densities on the fluorine atoms of **2** compared to **1** (Table S9).^27^

Hyperfine signals due to ^14^N were also observed in the HYSCORE spectra of **1** and **2** (Figures 7 and 8) and are characteristic of double-quantum (DQ) transitions (Δ*m_I_* = ±2). They appear in the strongly coupled (−, +) quadrant, when |*A*| > 2 ν*_N_*, and are centered at the hyperfine (*A*) and spread by four times the Larmor frequency (4ν*_N_*). The quadrupole coupling constant (*e* ^2^ *Qq/h*) can be estimated from the position of the ridges according to 5,

5 $$\upsilon_{\alpha ,\beta }^{DQ}=2\sqrt{{\left(\upsilon \pm \frac{A}{2}\right)}^{2}+{\left(\frac{e^{2}\mathrm{qQ}}{4\;h}\right)}^{2}\left(3+\eta^{2}\right)}$$

where ν^DQ^ are the frequencies of the double-quantum transitions, ν is the Larmor frequency of the corresponding nuclei and the other symbols have the usual meaning. Knowing that the asymmetry parameter’s lower and upper limits are 0 and 1, respectively, eq 5 gives a range of *e* ^2^ *Qq/h*.

**Figure 7:**
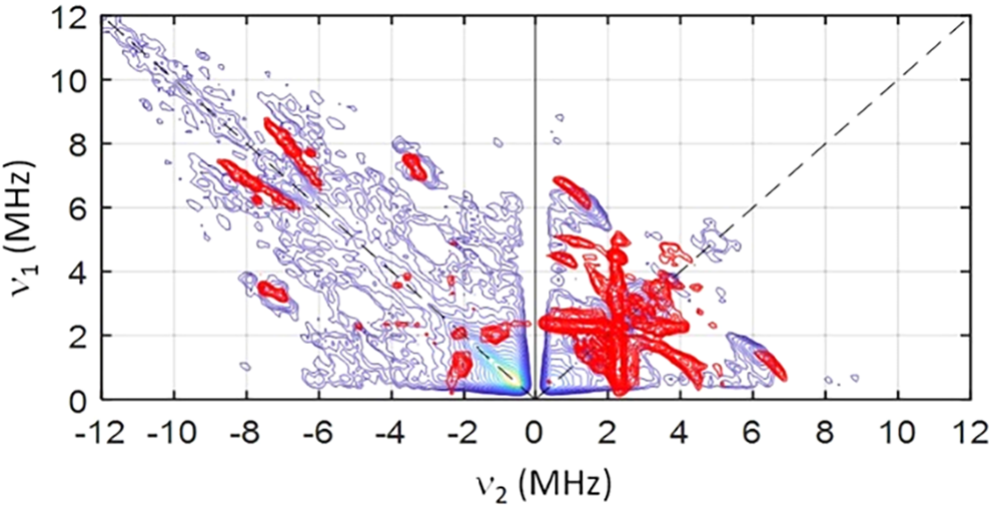
X-band (ca. 9.7 GHz) experimental (blue) and simulation (red) ^14^N, ^67^Zn-HYSCORE spectra of compound **1** at OP2 and τ = 200 ns. The black dashed lines mark the diagonal and antidiagonal where ν_1_ = ν_2_ and −ν_1_ = ν_2_.

**Figure 8:**
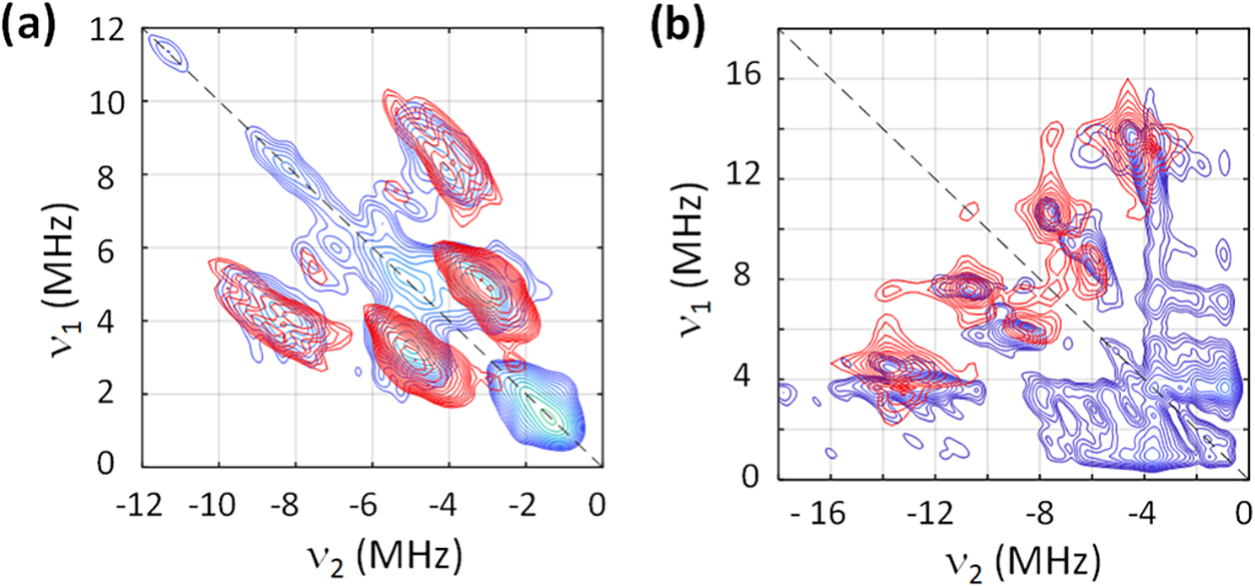
Experimental (blue) and simulation (red) ^1^N-HYSCORE spectra of compound **2** (OP1) at X-band (ca. 9.7 GHz), τ = 150 ns (a), and at Q-band (ca. 34 GHz), τ = 300 ns (b). The black dashed lines mark the antidiagonal, where −ν_1_ = ν_2_.

In compound **1**, the hyperfine ridges due to NIT ^14^N nuclei appear at around [−7.0, 3.0] and [−3.0, 7.0] MHz (Figure 7). They corroborate with the hyperfine interaction (HFI) values obtained by CW-EPR and can thus be modeled considering two equivalent nuclei and HFI coupling constants from Table 1, |*A*_*x*,*y*,*z*_ *^N^* | = 57, 5.2, 4.4 MHz, and orientation with respect to the molecular frame of [0, 15, 0] degrees in Euler angles (*zyz* convention). The simulation yields *e* ^2^ *Qq/h* = −2.5 MHz and η = 0.3. The same spin Hamiltonian (2) and parameters reproduce other observer positions (ESI Figure S15). It is also possible to detect signals at ca. [1.56, 6.0], [6.0, 1.56] MHz in Figure 7 corresponds to ^67^Zn in the weakly coupled region. These were simulated considering an HFI tensor of |*A*_*x*,*y*,*z*_^Zn^|= 1.4, 0.15, and 0.15 MHz and NQI parameters of *e* ^2^ *Qq/h* = 14 MHz and η = 0.6. Notably, the same parameters reproduce the other observer positions (ESI Figure S15). Large *e* ^2^ *Qq/h* values are reported for ^67^Zn in distorted octahedral coordination environments.^35,36^

The ^14^N signals were also observed in the HYSCORE spectra of **2**, as two quasi-equivalent nuclei give rise to double-quantum transition ridges at around [−8.3, 4.4], [−4.4, 8.3] MHz and single-quantum ones at [−4.88, 2.93], and [−2.93, 4.88] MHz (Figure 8a). These were well simulated with the HFI parameters *A*_*xyz*_^*N*1^ = −1, −1, and −17 MHz, and *A*_*z*_^*N*2^ = −18.7 MHz, and NQI parameters *e* ^2^ *Qq*_1_ */h* = −2.5 MHz, and η_1_ = 0.3, and *e* ^2^ *Qq*_2_ */h* = −2.3, and η_2_ = 0.1 in every observer position and τ-value measured (ESI Figure S16). To try to disentangle these signals, *Q*-band HYSCORE was also used, and the corresponding EDFS and simulation are provided in the ESI (Figure S17). This strategy is useful, since the Larmor frequency is field-dependent, while the other EPR parameters are not. Hence performing the same experiment in higher frequencies and magnetic fields may resolve overlapping transitions by shifting them from the strongly coupled to the weakly coupled quadrant. The signals are clearly observed in the (−, +) region and are well simulated with the same model as used for X-band data (Figures 8 and S18). These signals stem from the two nitrogen atoms in the nitronyl-nitroxide radical moiety. Notably, measured hyperfine couplings to ^14^N nuclei are three times weaker for **2** compared to **1**, which is exactly as expected. Even so, the magnitude of these couplings is significant, and thus one can safely assume that ^14^N and ^1^H nuclei of nitronyl-nitroxide radical moiety contribute to spin relaxation and decoherence in both **1** and **2**, though the strongest effect is expected for **1**.

### ENDOR

Electron nuclear double resonance spectroscopy was also used to evaluate the strength of hyperfine couplings and corroborate the HYSCORE findings. As anticipated, Mims ENDOR spectra of **1** and **2** show clear contributions from ^1^H and ^19^F nuclei (Figure 9). Data for compound **1** are in full agreement with HYSCORE data (Figures S20 and S21). In the case of compound **2**, ENDOR spectra could only capture the smallest interactions from hfac fluorines F39,40,41, proton H38, and the methyl protons H54,52,53 (Figure S22).

**Figure 9:**
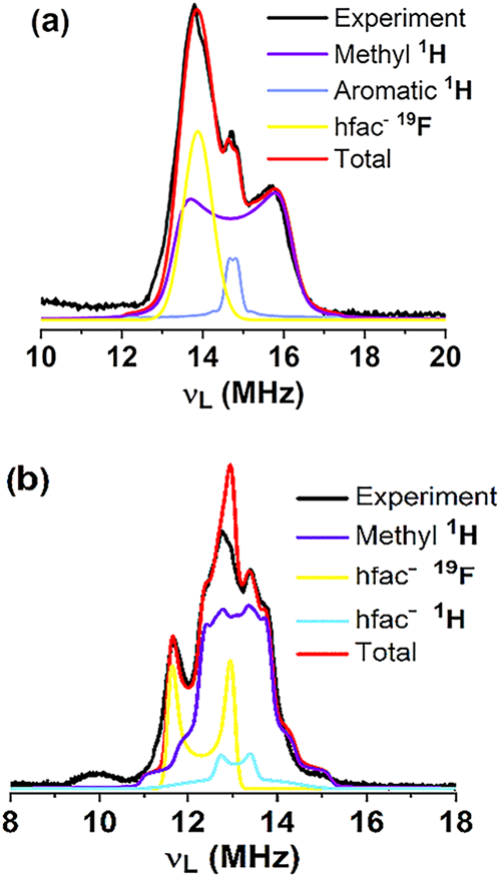
X-band (ca. 9.7 GHz) experimental (black) and simulations (see legend) ^1^H, ^19^F Mims ENDOR spectra of a 1.2 mmol·L^–1^ solution of (a) compound **1** at 9 K, 347 mT and τ = 200 ns and (b) compound **2** at 5.5 K, 307.6 mT and τ = 300 ns.

## Conclusions

The qubit behavior of two mononuclear complexes has been investigated: (Et_3_NH)­[M­(hfac)_2_L] (M = Zn **1**, Ni **2**). Both compounds are characterized by the *S* = 1/2 ground state (below 120 K for the nickel derivative). For compound **1**, the spin density is located mainly on the radical, while for complex **2**, the bulk density is on nickel. The anisotropic g tensor [2.26 2.28 2.32] implies significant SOC, which affects the electron spin relaxation properties. At elevated temperatures, the nickel complex shows much shorter relaxation times than the zinc one. For the zinc complex, the phase memory time, *T*_m_, is almost temperature-independent (ca. 1.50 μs) below 80 K. Its spin–lattice relaxation time, *T*_1_, reaches 4.1 ms at 5 K, and is still 0.2 ms at 100 K. In contrast, *T*_1_ for **2** shows a stronger temperature dependency decreasing from 0.9 ms at 5.5 K to only 0.3 μs at 100 K. As a consequence, *T*_m_ for **2** is limited to 0.12 μs at 100 K. At low temperatures, *T*_m_ for **2** (3.7 μs at 5.5 K) is longer than that of **1** (1.78 μs at 5.2 K) suggesting that hyperfine couplings to ligand nuclei play a more dominant role in spin relaxation.^14b^ Use of a CPMG pulse sequence to reduce ESEEM effects has prolonged *T*_m_ up to 18 μs for **1**, 7 μs for **2** and 5.5 K. Compared to *T*_m_, there is a 10-fold increase in the relaxation time of **1** and 2-fold for **2** (Tables S5 and S6). Transient nutation experiments revealed coherent Rabi oscillations for both compounds. HYSCORE and ENDOR spectroscopies show clear evidence of ^1^H, ^14^N, ^19^F, and ^67^Zn participating to spin decoherence; accidentally or not, **2** shows stronger ^19^F coupling (electron density on nickel) and thus faster decoherence. In the case of compound **1**, electronic density is only little delocalized toward ^19^F, though there is spin density on zinc as ^67^Zn hyperfine coupling is observed. ^19^F coupling in **1** is well modeled with dipolar contribution only.

Further work on systems constructed from homo- and heterobiradicals and their complexes is in progress.

## Experimental Section

The synthesis and crystal structures of **1** and **2** were previously reported.^27^ CW EPR measurements were performed on a Bruker EMXplus EPR spectrometer operating at X- (ca. 9.5 GHz) or Q-band (ca. 34 GHz), and at variable temperatures. Solution spectra were recorded in dichloromethane/toluene (9:1 v/v) mixtures (1.3 and 5 mM), while powder spectra were measured on finely ground crystals. Field corrections were applied in all cases against a Bruker strong pitch reference sample (*g* = 2.0028). Pulse EPR measurements were performed on frozen solution samples (0.5 mM) on a Bruker ELEXSYS E580 spectrometer operating at X- (ca. 9.7 GHz) or Q-band (ca. 34 GHz). Cryogenic temperatures were achieved using a cryogen-free closed-cycle helium circuit. Echo-detected field swept (EDFS) spectra were acquired with a two-pulse, Hahn-echo sequence (π/2−τ–π–τ–echo),^33^ under fixed interpulse delay, τ, and with varying the static magnetic field. The measurements were obtained from dichloromethane/toluene (9:1 v/v) solutions, and the time-domain experiments were done in different magnetic fields (observer positions). Phase memory times were measured at selected field positions by varying τ in the standard Hahn sequence, and long (selective) pulses were necessary to suppress modulation effects from proton and ^14^N nuclei. Carr–Purcell–Meiboom–Gill (CPMG) spin–spin relaxation time measurements were carried out using a π/2–(τ–π–τ–echo)*_n_* sequence, with τ = 300 and *n* = 398. The curves were fitted with the biexponential curve in eq 6 unless otherwise stated.

6 $$Y\left(t\right)=Y_{f}\exp\left(-\frac{t}{T_{f}}\right)+Y_{s}\exp\left(-\frac{t}{T_{s}}\right)$$

where the subscripts f and s stand for fast and slow, respectively, and the other symbols have their usual meaning. The fast component can be attributed to some molecules that have spin–spin interaction with neighbors, which may occur in randomly diluted systems.^19a^

Spin–lattice relaxation times were measured using an inversion recovery sequence (π–*t*–π/2−τ–π–τ–echo) and varying the interpulse delay *t* at a fixed magnetic field. The curves were also fitted with the biexponential eq 6, where the fast component is attributed to spectral diffusing, *T*_SD_, which is commonly 1 order of magnitude smaller than *T*_1_.

Rabi oscillations were detected using a transient nutation pulse sequence (*t*_p_–*t*–π/2−τ–π–τ echo) and varying the tipping pulse length, *t*_p_. The oscillation curves were baselined with a polynomial function, and the Rabi frequency, Ω_R_, was determined by applying the Fourier Transform. Relative *B*_1_ was calculated according to 7

7 $$B_{1}=\sqrt{\frac{10^{-0.1a}}{10^{-2.4}}}$$

where *a* is the microwave attenuation in dB.

Hyperfine sublevel correlation (HYSCORE) spectroscopy was performed using a four-pulse sequence (π/2τ- π/2-*t*_1_ -π -*t*_2-_ π/2- τecho) with π/2 pulses of 16 ns and fixed τ. The intervals *t*_1_ and *t*_2_ started at 100 ns and were incremented independently to give a 2D correlation pattern. The acquired time-domain signal was background corrected with polynomial functions, zero-filled to 1024 points, apodized with Hamming window and Fourier transformed to give the frequency-domain spectra.

Electron nuclear double resonance (ENDOR) data were acquired using the Mims sequence (π/2−τ–π/2–RF–π/2–echo) with π/2 pulses of 16 ns and fixed τ. Radiofrequency (RF) pulses of 14 μs were used.

Both experiments were performed at different τ-values to avoid blind spot effects inherent to ESEEM and Mims ENDOR spectroscopies. The EasySpin package was used to simulate the data.^29^

The dipolar hyperfine contributions were calculated using the point-dipole model eq 8,

8 $$A_{dip}=\frac{\mu_{0}}{4\pi h}\mu_{B}\mu_{n}\sum_{k}\rho k\frac{3\left(gn_{k}\right)\left(n_{k}^{T}g_{n}\right)-gg_{n}}{r_{k}^{3}}$$

where the summation is over all atoms *k* carrying spin density, *n* is the electron–nuclear unit vector, *n^T^* its transpose, *g_n_* is the nuclear *g* diagonal matrix, ρ is the electron spin population at the atom *k*, *r*_*k*_ is the distance between electron and nucleus *k*, and other symbols have the usual meaning. The ρ values were obtained by normalizing to 1 (100%) the sum of the spin densities determined by DFT.

EDFS, Hahn-echo decay, CPMG, and nutation experiments were done in 0.5 mmol·L^–1^ solutions.

No uncommon hazards are noted.

## Supplementary Material



## Notes

CW-EPR spectra, echo decay, inversion recovery, and CPMG curves, Rabi oscillations, HYSCORE and ENDOR spectra (PDF)

## Notes

This paper is dedicated to Dr. Luminiţa Patron on the occasion of her 80th anniversary.
