# Supplementary material for: Investigation of Hyperfine Interactions in Molecular Spin Qubits Constructed from A Nitronyl-Nitroxide Ligand and Transition Metal Ions
Source: Inorg Chem. 2026 Mar 13;65(12):6544–53. doi: 10.1021/acs.inorgchem.5c05585 (PMC13040534; doi:10.1021/acs.inorgchem.5c05585)

Electronic Supporting Information

Investigation of hyperfine interactions in molecular spin qubits constructed from a nitronyl-nitroxide ligand and transition metal ions

Daniel O. T. A. Martins[†], Cristian A. Spinu[‡,§], Alena Sheveleva,[†] Mihaela Hillebrand,[‡] Floriana Tuna*[†], Marius Andruh*[‡,§]

[†] *Department of Chemistry and Photon Science Institute, University of Manchester, Oxford Road, Manchester, M13 9PL, UK*
[‡] *Faculty of Chemistry, University of Bucharest, Regina Elisabeta Blvd. 4-12, Bucharest – 030018, Romania.*
[§] *C. D. Nenitzescu Institute of Organic and Supramolecular Chemistry of the Romanian Academy, Splaiul Independentei, 202B, 060023, Bucharest, Romania*
*email: Floriana.Tuna@manchester.ac.uk; marius.andruh@acad.ro.

Table of contents

CW-EPR spectra 2
Relaxation Times 4
CMPG Measurements 7
Rabi Oscillations 8
Dipolar hyperfine calculation 10
HYSCORE 12
ENDOR 16
Mulliken Spin densities 17

CW-EPR spectra

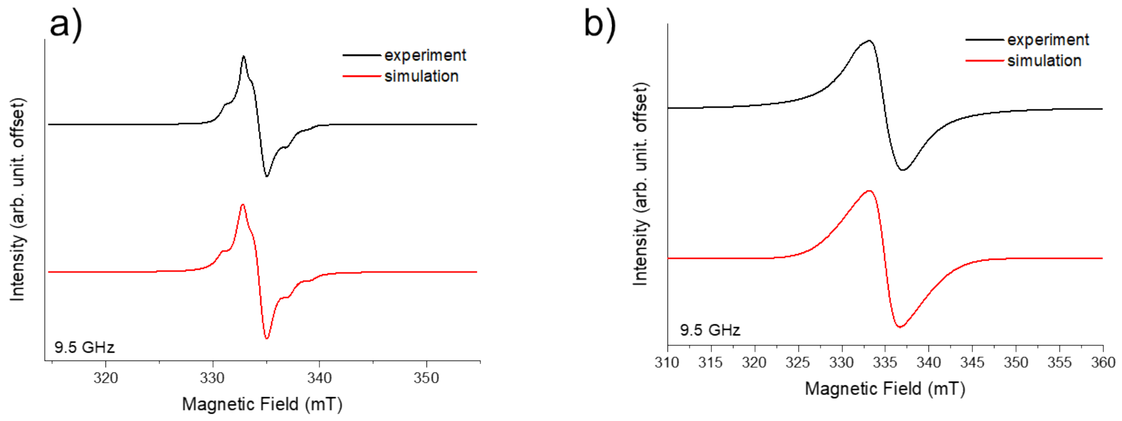

Figure S1. Experimental (black) and simulated (red) X-band EPR spectra of **1**: (a) frozen solution; (b) powder. Simulation parameters are provided in **Error! Reference source not found.**.

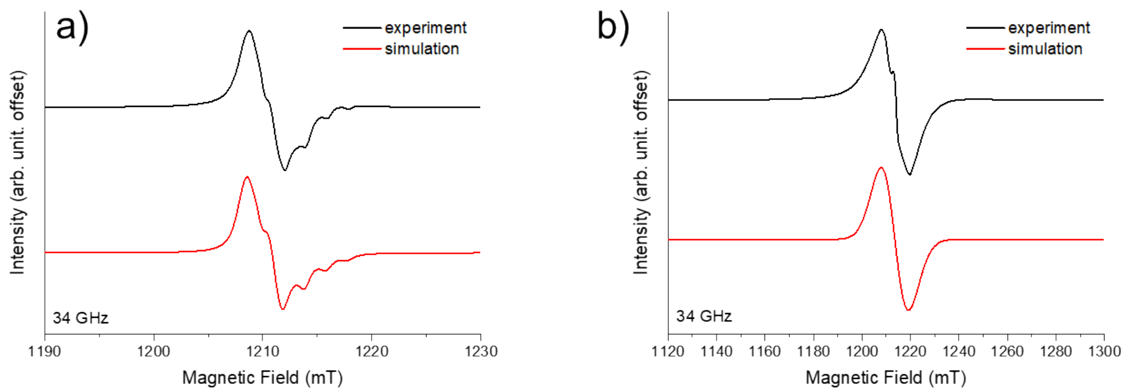

Figure S2. Experimental (black) and simulated (red) Q-band EPR spectra of **1**: (a) frozen solution, abd (b) powder. Simulation parameters are provided in **Error! Reference source not found.**.

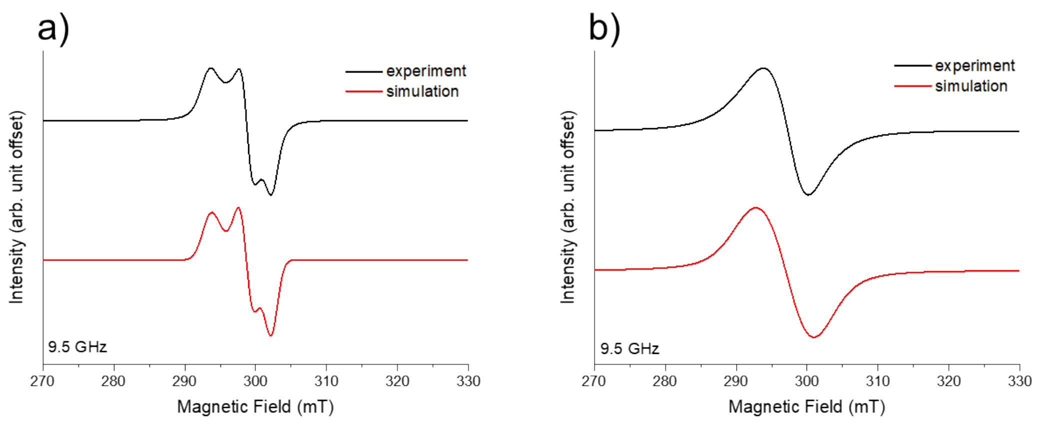

Figure S3. Experimental (black) and simulated (red) X-band EPR spectra of **2**: (a) frozen solution, and (b) powder. (b). Simulation parameters are listed in **Error! Reference source not found.**.

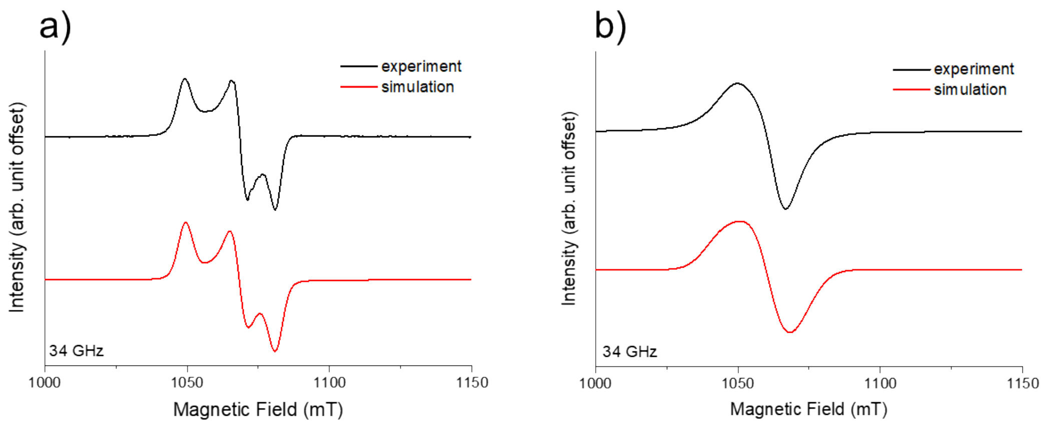

Figure S4. Experimental (black) and simulated (red) Q-band EPR spectra of **2: (a)** frozen solution, and (b) powder. Simulation parameters are provided in **Error! Reference source not found.**.

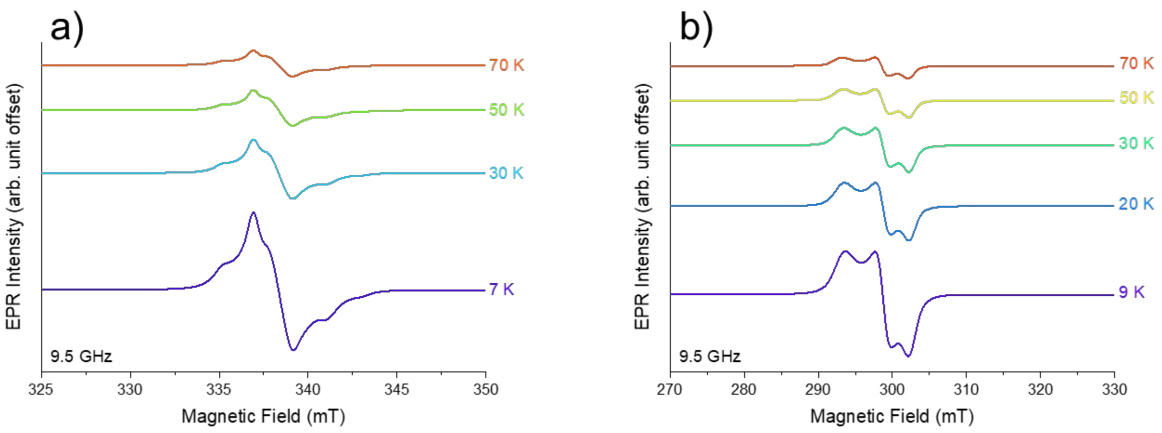

Figure S5. Variable tempersature X-band EPR spectra of a 1.3 mM solution of a) **1** and b) **2**.

Relaxation Times

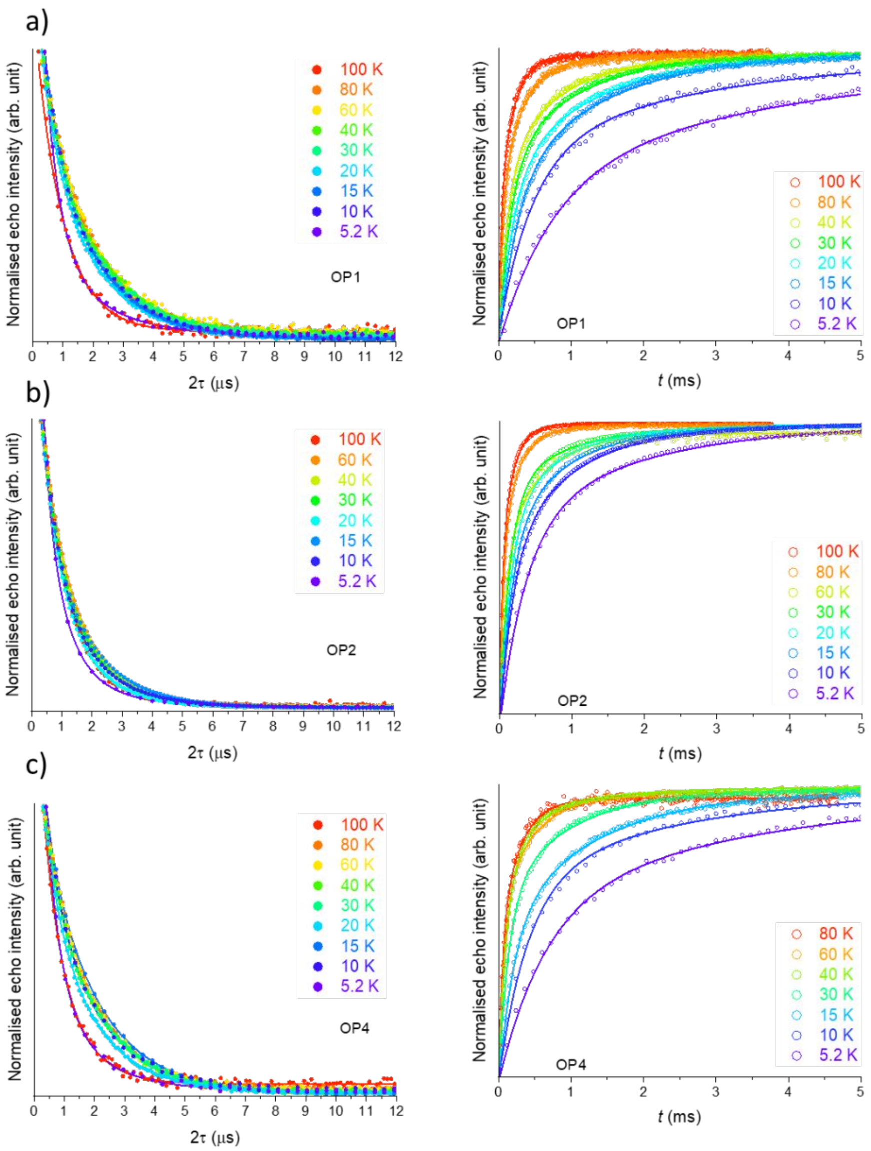

Figure S6. Normalized Hahn echo intensities as a function of the interpulse delay 2*τ* (left), and inversion recovery curves (right) for a frozen solution of 1 (0.5 mM) at X-band and temperatures from 5.2 to 100 K, at (a) OP1; (b) OP2 and (c) OP4 (based on Figure 2 a)). The solid lines are fits to the bi-exponential equation, with parameters in Tables S2 and S3.

Table S1. Extracted spin-lattice relaxation time constants of **1** from the fit to the bi-exponential equation.

	OP1		OP2		OP4	
T (K)	T_1 (μs)	T_{SD}	T_1 (μs)	T_{SD} (μs)	T_1 (μs)	T_{SD} (μs)
**5.2**	4 146(20)	744(5)	1825(7)	365(1)	3 525(16)	641(4)
**10**	2 719 (18)	461(4)	971 (2)	184.5(7)	2 374(14)	407(3)
**15**	1 403(4)	257(1)	909(3)	176.6(7)	1 300(4)	212(1)
**20**	1 333(4)	211(1)	859(4)	160.6(7)		
**30**	1 075(4)	170.4(8)	948(6)	148.5(6)	964(4)	147.7(7)
**40**	828(3)	131.0(7)			763(5)	122.6(5)
**60**			546(3)	88.5(8)	511(3)	84.5(6)
**80**	324(1)	55.0(1)	250.1(8)	51.5(3)	279(2)	50.6(6)
**100**	197.6(7)	35.0(3)	169.8(4)	37.6(2)		

Table S2. Extracted phase memory time constants of **1** from the fit to the bi-exponential equation.

	OP1		OP2		OP4	
T (K)	T_m (μs)	$T_{m,fast}$	T_m (μs)	$T_{m,fast}$	T_m (μs)	$T_{m,fast}$
**5.2**	1.78(5)	0.50(1)	1.38(1)	0.442(3)	1.50(4)	0.46(1)
**10**	1.67(1)	0.44(2)	1.490(3)	0.516(2)	1.56(2)	0.40(3)
**15**	1.702(5)	0.511(8)	1.554(2)	0.539(2)	1.712(6)	0.33(2)
**20**	1.637(7)	0.48(1)	1.304(9)	0.600(5)	1.582(7)	0.53(1)
**30**	1.71(1)	0.51(2)	1.361(8)	0.629(7)	1.66(7)	0.47(1)
**40**	1.72(1)	0.47(2)	1.43(1)	0.70(1)	1.637(9)	0.52(2)
**60**	1.61(2)	0.6(1)	1.55(4)	0.88(1)	1.53(2)	0.7(1)
**80**	1.49(1)	0.3(1)			1.44(3)	0.9(10)
**100**	0.927(5)	0.5(1)	1.02(1)	0.47(1)	0.95(4)	0.46(7)

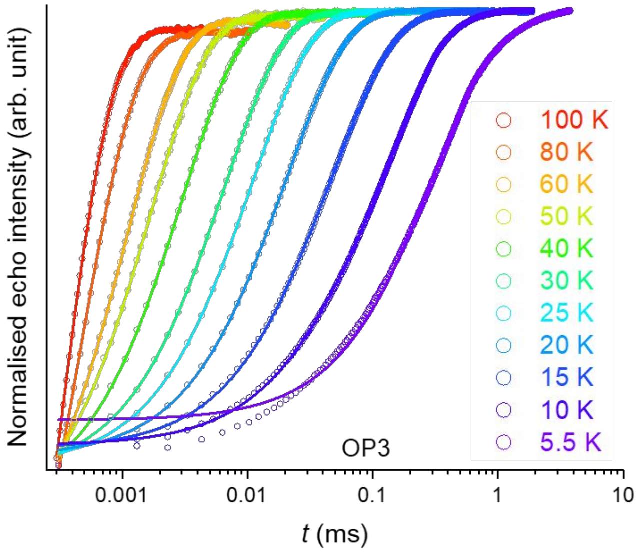

Figure S7. Inversion recovery curves at OP3 (Figure 2b) for a 0.5 mM solution of **2** at temperatures between 5.5 and 100 K. Solid lines are the fits to the bi-exponential equation, with parameters in Table S4.

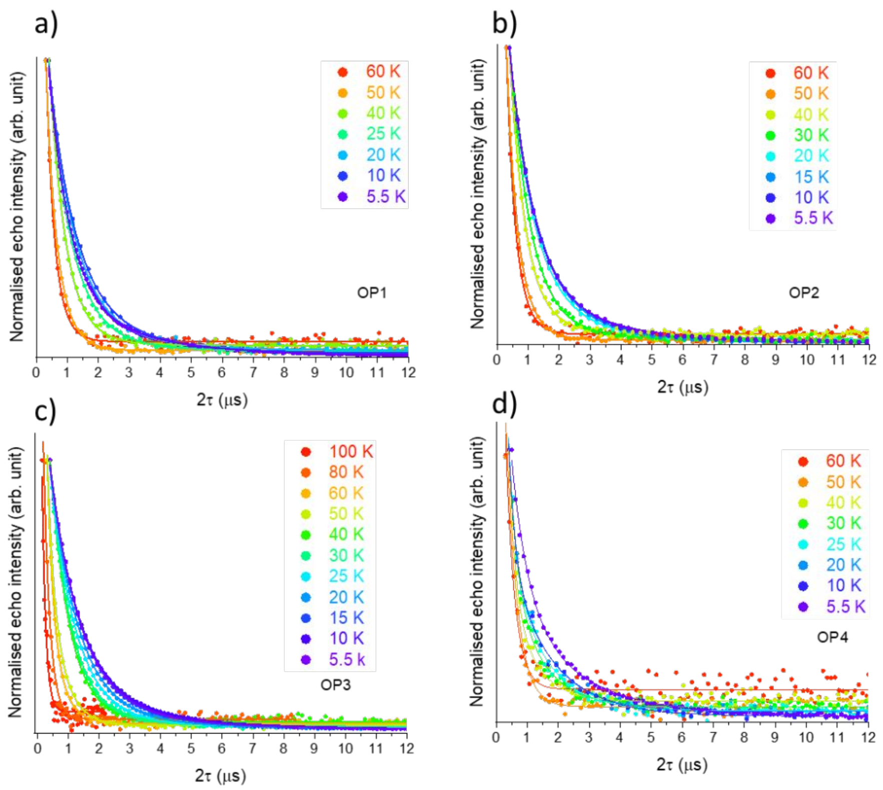

Figure S8. Normalised Hahn echo intensities as a function of the interpulse delay 2τ of a 0.5 mM solution of **2** at temperatures between 5.5 and 100 K, and at different observer positions: (a) OP1, (b) OP2, (c) OP3 and (d) OP4, based on Figure 2b. Solid lines are the fits to the biexponential equation, with parameters in Table S5.

Table S3. Extracted spin-lattice relaxation time constants of **2** from the fit to the bi-exponential equation

OP3		
T (K)	T_1 (μs)	T_{SD} (μs)
**5.5**	908(9)	258.1(8)
**10**	151.6(1)	25.6(2)
**15**	62.74(6)	20.2(1)
**20**	25.61(4)	9.95(7)
**25**	13.00(3)	4.8(1)
**30**	7.59(2)	2.77(8)
**40**	3.50(2)	1.7(1)
**50**	2.07(6)	1.26(8)
**60**	1.098(3)	0.07(4)
**80**	0.8(3)	0.44(3)
**100**	0.3(1)	-

Table S4. Extracted phase memory time constants of **2** from the fit to the bi-exponential equation

	OP1		OP2		OP3		OP4	
T (K)	T_m (μs)	$T_{m,fast}$	T_m (μs)	$T_{m,fast}$	T_m (μs)	$T_{m,fast}$	T_m (μs)	$T_{m,fast}$
**5.5**	2.62(3)	0.67(1)	2.46(4)	0.766(6)	3.7(1)	0.985(4)	2.25(2)	0.548(7)
**10**	2.17(8)	0.82(1)	2.21(3)	0.783(6)	3.39(4)	0.965(3)	1.41(2)	0.206(7)
**15**			2.02(3)	0.777(6)	3.35(6)	0.978(3)		
**20**	2.1(2)	0.82(2)	1.70(7)	0.72(1)	3.36(9)	0.913(3)	1.21(2)	0.18(1)
**25**	2.0(1)	0.69(1)			3.2(2)	0.825(4)	1.07(2)	0.21(1)
**30**			1.8(2)	0.66(1)	3.0(2)	0.698(3)	1.0(4)	0.24(1)
**40**	1.8(7)	0.52(1)	1.1(2)	0.48(2)	0.640(3)	0.19(1)	0.78(5)	0.19(2)
**50**	0.42(1)	0.18(3)	0.51(2)	0.27(2)	0.50(1)	0.19(1)	0.36(1)	-
**60**	0.35(8)	0.20(8)	0.34(2)	0.16(6)	0.44(3)	0.19(1)	0.29(1)	-
**80**	0.23(3)	0.018(9)			0.205(3)	-	0.18(4)	-

**100**					0.118(2)	-		

CPMG Measurements

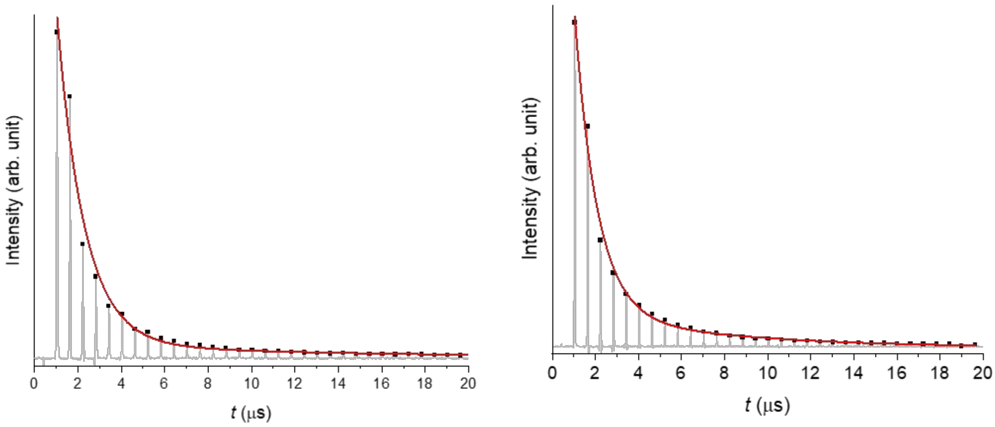

Figure S9. Echo intensities from the CPMG sequence at 5.5 K as a function of the interpulse delay (grey lines), absolute values (black scatter), and corresponding fit to the bi-exponential equation (red line) for **1** at OP2 (a) and **2** at OP3 (b).

Table S5. Extracted CPMG time constants of **1** from the fit to the bi-exponential equation

	OP1		OP2		OP4	
**T (K)**	T_{CPMG} (μs)	$T_{\mathrm{CPMG,fast}}$	T_{CPMG} (μs)	$T_{\mathrm{CPMG,fast}}$	T_{CPMG} (μs)	$T_{\mathrm{CPMG,fast}}$
**5.5**	13(2)	1.5(1)	15(3)	1.2(1)	18(1)	1.4(1)
**15**	17(3)	2.7(4)	11(2)	1.8(2)		
**30**	13(1)	3.2(5)	10(1)	2.0(3)	12(1)	2.7(5)
**60**	14(1)	3.4(6)	9(1)	2.1(2)	13(2)	2.9(7)
**80**					10(2)	2.6(6)

Table S6. Extracted CPMG time constants of **2** from the fit to the bi-exponential equation

	OP1		OP2		OP3		OP4	
**T (K)**	T_{CPMG} (μs)	$T_{\mathrm{CPMG,fast}}$	T_{CPMG} (μs)	$T_{\mathrm{CPMG,fast}}$	T_{CPMG} (μs)	$T_{\mathrm{CPMG,fast}}$	T_{CPMG} (μs)	$T_{\mathrm{CPMG,fast}}$
**5.5**	6(1)	1.46(6)	6(1)	1.20(8)	7(7)	1.05(9)	4.1(8)	1.01(4)
**15**	8(4)	1.6(1)	5(2)	1.1(1)	5(4)	1.00(8)	2.2(3)	0.9(1)
**30**	2(1)	1.1(3)	3(1)	1.0(1)	4(2)	0.89(5)	1.08(3)	1.0(4)

Rabi Oscillations

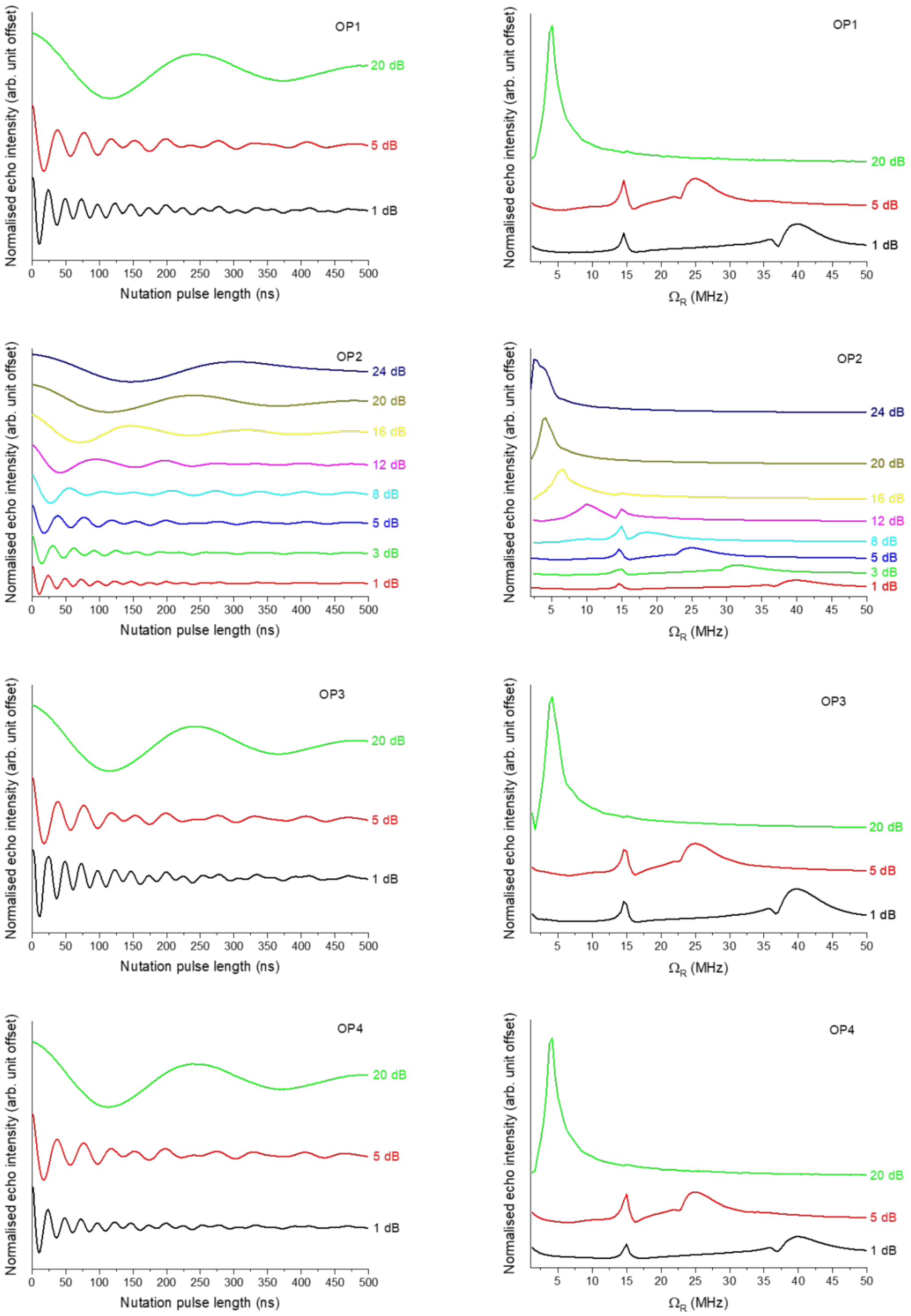

Figure S10. Rabi oscillations acquired with different microwave attenuations (left) and corresponding Fourier transforms (right) for 0.5 mM solution of **1** at different observer positions. The peak at around 15 MHz corresponds to the Larmor frequency of the ^{1}H nucleus.

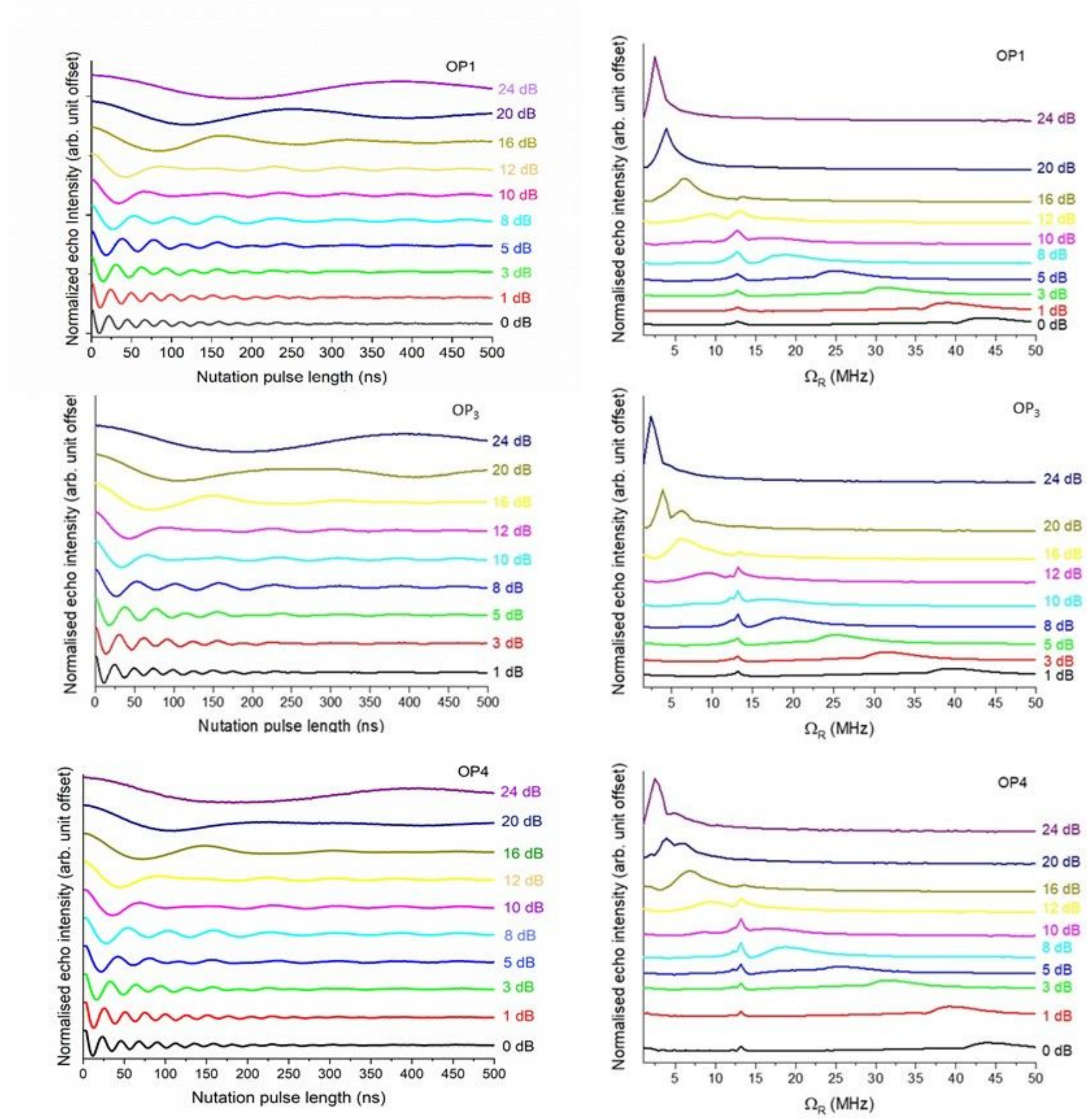

Figure S11. Rabi oscillations acquired with different microwave attenuations (left) and corresponding Fourier transforms (right) for a 0.5 mM solution of compound **2** at different observer positions. The peak at around 15 MHz corresponds to the Larmor frequency of the ^{1}H nucleus.

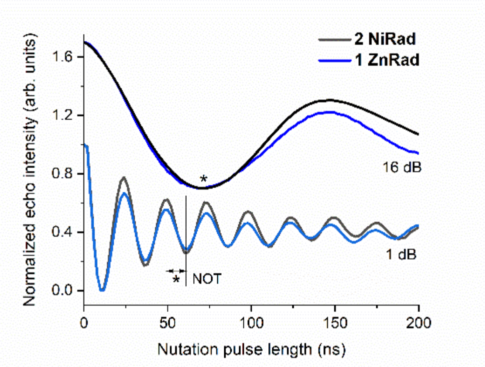

Figure S12. Comparison of Rabi oscilations for **1** (blue) and **2** (black) acquired at mw attenuation of 16 db (top) and 1 dB (bottom) at 10 K, highligtuing the NOT operation times (*).

Dipolar hyperfine calculation

Table S7. Dipolar hyperfine contributions for **1**, calculated with the point-dipole model, and the isotropic hyperfines from HYSCORE and ENDOR simulations.

Atoms	Label	A_{dip} *(MHz)* $\begin{bmatrix} xx & xy & xz \\ yx & yy & yz \\ zx & zy & zz \end{bmatrix}$	A_{dip} *(MHz)* *x, y, z*	***Euler angles zyz (deg)*** *α,β, γ*	A_{iso} *(MHz)*
**Methyl protons**	**H52,53,54**	-1.7266 0.2143 -0.0766<br>0.2138 0.8346 -2.8876<br>-0.0763 -2.8815 0.8866	3.7529<br>- 1.7042<br>-2.0542	161.8928<br>131.7306<br>-113.1270	-0.3
	**H55,56,57**	-1.9015 -0.0451 0.8361<br>-0.0450 -1.2261 2.4475<br>0.8326 2.4423 3.1172	4.3048<br>-1.7422<br>-2.5731	-8.1148<br>66.5818<br>-53.6891	
	**H62,63,64**	0.4285 3.1601 -0.3233<br>3.1535 2.2082 -0.8119<br>-0.3219 -0.8102 -2.6303	4.6949<br>-1.8973<br>-2.7912	-66.7509<br>163.1775<br>-120.7472	
	**H65,66,67**	0.8103 -0.7987 -2.8409<br>-0.7970 -1.9514 0.8915<br>-2.8291 0.8896 1.1404	4.0531<br>-1.8617<br>-2.1921	43.1595<br>101.7884<br>93.8934	
**Aromatic *ortho* proton**	**H45**	-2.1051 -0.2240 0.0418<br>-0.2236 -2.0585 1.6187<br>0.0416 1.6154 4.1505	-1.9931<br>-2.5664<br>4.5464	-116.6764<br>166.2381<br>-90.4552	2.0(2)
***hfac⁻* fluorine**	**F31,32,33**	-0.0220 0.2129 0.3184<br>0.2125 -0.0943 0.2651<br>0.3171 0.2646 0.1160	0.5510<br>-0.2782<br>-0.2731	-43.8993<br>106.9887<br>66.8834	0
	**F35,36,37**	0.1726 -0.2264 0.2691<br>-0.2259 -0.1150 -0.1512<br>0.2680 -0.1509 -0.0571	0.4751<br>-0.2340<br>-0.2406	-123.2179<br>113.8692<br>-75.9139	
	**F38,39,40**	0.1167 -0.1332 -0.2932<br>-0.1329 -0.1697 0.1133<br>-0.2920 0.1131 0.0532	0.4301<br>-0.2078<br>-0.2221	-129.8884<br>85.9695<br>114.7002	
	**F42,43,44**	0.1844 0.0855 -0.0526<br>0.0853 -0.0845 -0.0166<br>-0.0524 -0.0165 -0.0993	0.2188<br>-0.1078<br>-0.1103	102.5620<br>128.3432<br>-98.3719	

Table S8. Dipolar hyperfine contributions for **2**, calculated with the point-dipole model, and the isotropic hyperfines from HYSCORE and ENDOR simulations.

Atoms	Label	A_{dip} *(MHz)* $\begin{bmatrix} xx & xy & xz \\ yx & yy & yz \\ zx & zy & zz \end{bmatrix}$	A_{dip} *(MHz)* *x, y, z*	***Euler angles zyz (deg)*** *α,β, γ*	A_{iso} *(MHz)*
**Methyl protons**	**H48,49,60**	-1.0182 0.5526 1.1015<br>0.5595 1.8436 0.4474<br>1.1348 0.4553 -0.8270	-2.0477<br>-0.0630<br>2.1091	46.4017<br>76.1003<br>105.0952	0.6
	**H51,52,53**	-2.7558 0.2186 -0.9289<br>0.2213 3.6512 -0.1551<br>-0.9570 -0.1578 -0.8761	-3.1503<br>-0.4973<br>3.6668	67.5405<br>87.5398<br>-87.6842	
	**H58,59,60**	0.8026 -0.1367 -0.3425<br>-0.1384 0.4193 -1.1875<br>-0.3529 -1.2084 -1.2537	-1.9288<br>0.8458<br>1.0514	-1.8975<br>118.3803<br>102.4269	
	**H61,62,63**	0.4095 1.0097 -0.2279<br>1.0223 -0.9565 0.5188<br>-0.2348 0.5279 0.5514	-1.6460<br>0.9572<br>0.6932	149.4567<br>162.8992<br>-149.5836	

***hfac*$^-$ proton**	**H38**	2.6185 -1.5897 -0.4753<br>-1.6096 -1.1403 0.1610<br>-0.4897 0.1639 -1.5375	3.2610<br>-1.7303<br>-1.5900	43.8186<br>171.6336<br>64.3347	2.0
	**H31**	-1.4396 -0.8075 0.0958<br>-0.8175 2.9626 -0.5700<br>0.0987 -0.5800 -1.5315	3.1799<br>-1.5834<br>-1.6051	-115.9457<br>163.7108<br>-35.1462	2.0
***hfac*$^-$ fluorine**	**F32,33,34**	0.2154 -1.0391 0.7656<br>-1.0521 0.0511 -0.7385<br>0.7888 -0.7515 -0.2738	1.7535<br>-0.9196<br>-0.8413	-23.1487<br>149.3167<br>-163.4747	-0.5
	**F35,36,37**	0.3752 -1.0320 0.8754<br>-1.0449 -0.2035 -0.6733<br>0.9019 -0.6852 -0.1795	1.7967<br>-0.9922<br>-0.8124	-1.7386<br>150.4709<br>-144.7549	2.0
	**F39,40,41**	0.3977 -0.2532 -1.3290<br>-0.2564 -0.8787 0.2155<br>-1.3692 0.2193 0.4844	1.8317<br>-0.9462<br>-0.8821	-151.8784<br>126.1689<br>52.2109	4.5
	**F42,43,44**	-0.5685 0.8698 0.2748<br>0.8807 1.2502 0.6998<br>0.2832 0.7121 -0.6865	1.8317<br>-0.9240<br>-0.9125	58.2500<br>146.6406<br>174.6045	2.0

HYSCORE

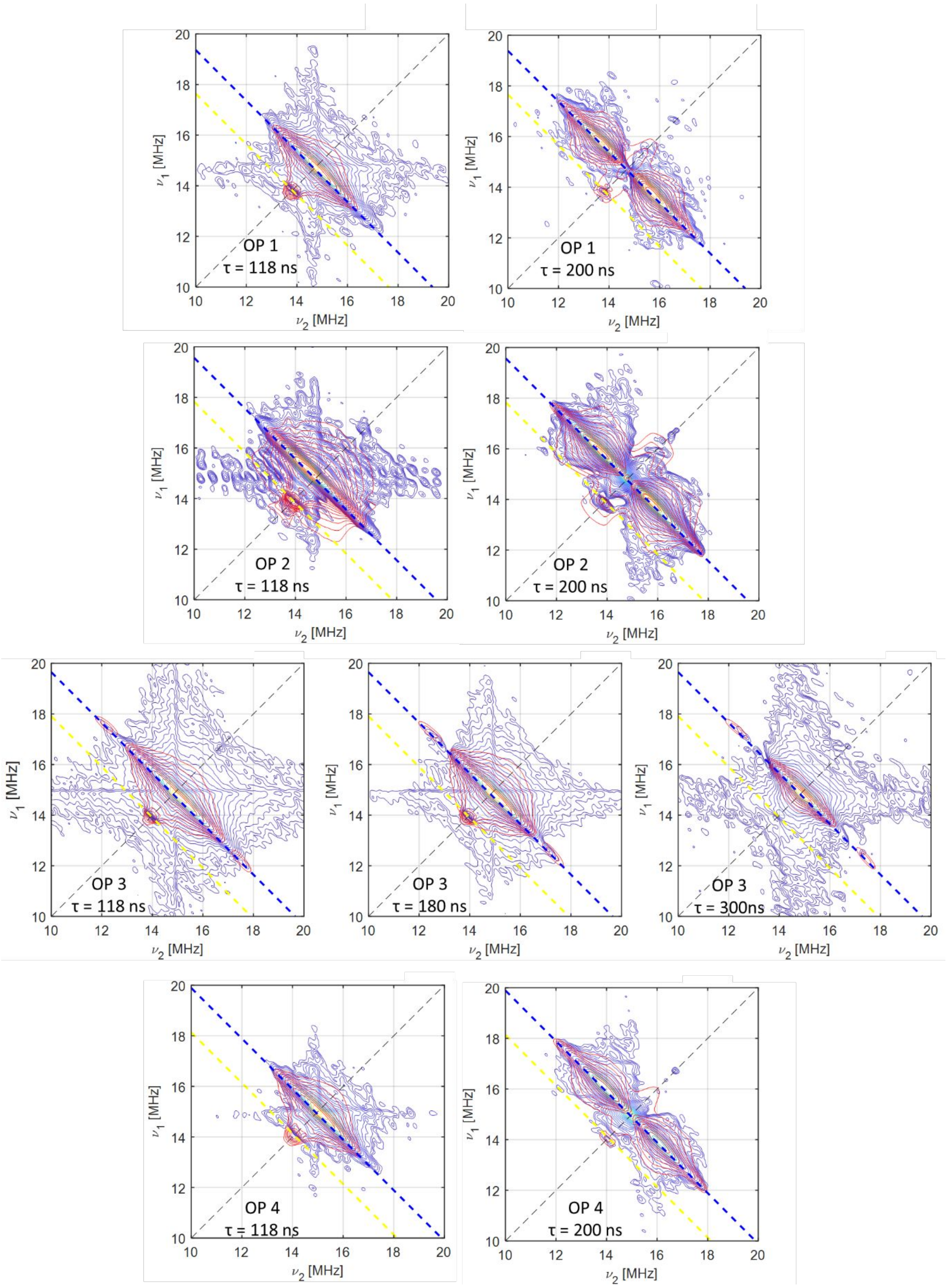

Figure S13. X-band (*ca.* 9.7 GHz) experimental (blue) and simulation (red) ^{1}H, ^{19}F-HYSCORE spectra of **1** at 5 K and different observer positions and interpulse delays, τ. The black dashed line marks the diagonal where $\nu_1 = \nu_2$. The blue and yellow dashed lines mark the Larmor frequencies of ^{1}H and ^{19}F respectively. Simulation parameters are provided in Table S7.

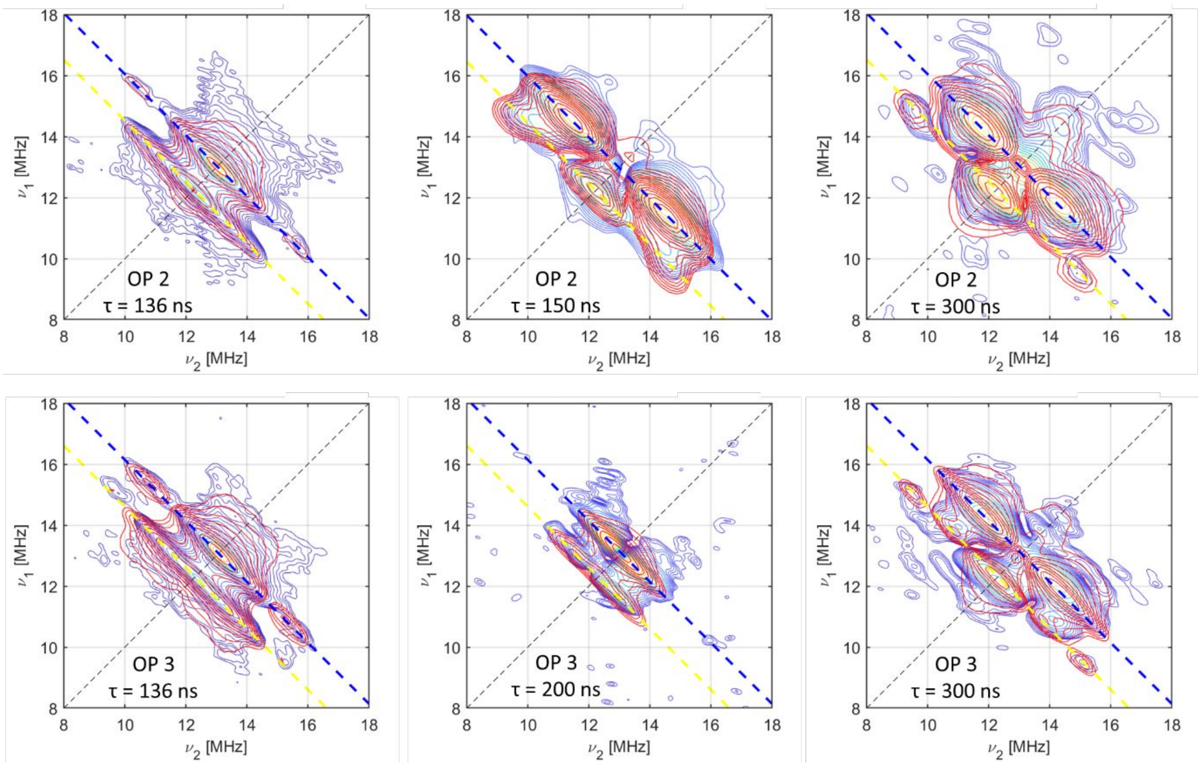

Figure S14. X-band (*ca.* 9.7 GHz) experimental (blue) and simulation (red) ^{1}H, ^{19}F-HYSCORE spectra of **2** at 5 K at different observer positions and interpulse delays, τ. The black dashed line marks the diagonal where $\nu_1 = \nu_2$. The blue and yellow dashed lines mark the Larmor frequencies of ^{1}H and ^{19}F respectively. Simulation parameters are presented in Table S8.

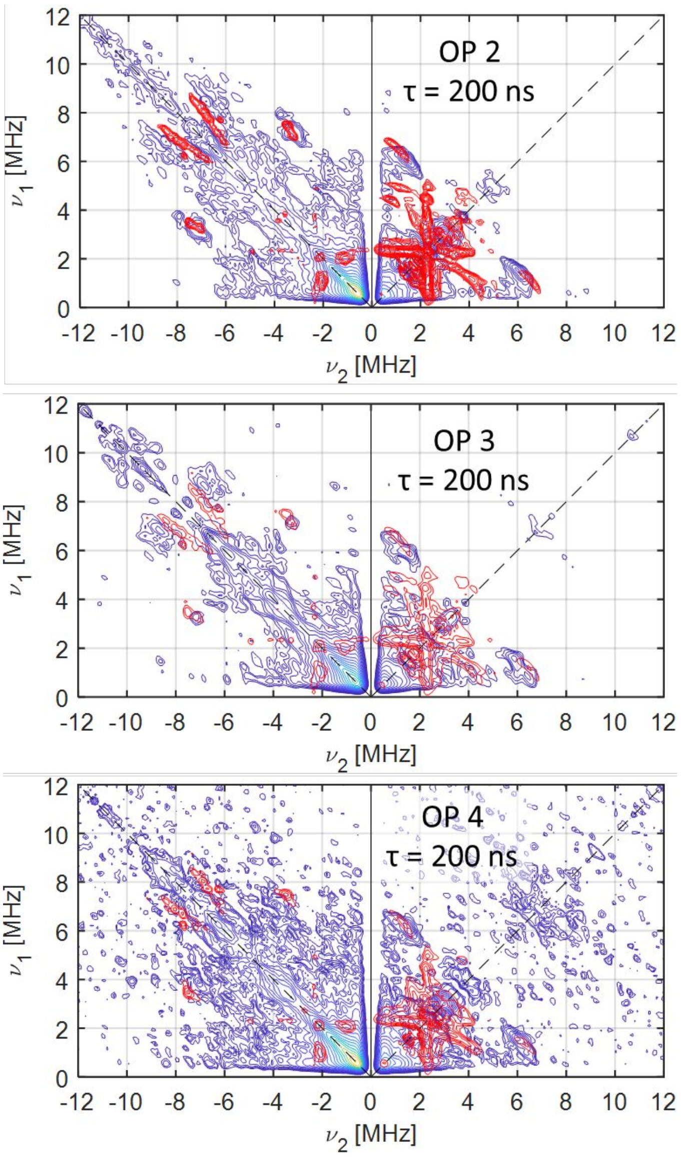

Figure S15. X-band (*ca.* 9.7 GHz) experimental (blue) and simulation (red) ^{14}N, ^{67}Zn-HYSCORE spectra of **1** at τ = 200 ns and different observer positions. The black dashed lines mark the diagonal and antidiagonal where $\nu_1 = \nu_2$ and $-\nu_1 = \nu_2$.

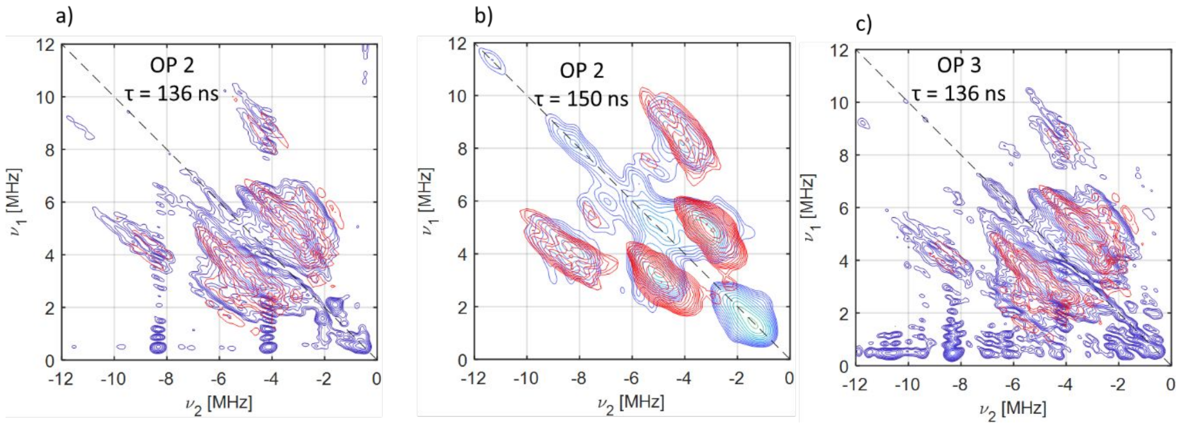

Figure S16. X-band (*ca.* 9.7 GHz) experimental (blue) and simulation (red) ^{14}N-HYSCORE spectra of **2** at different observer positions and interpulse delays. The black dashed line marks the antidiagonal where $-\nu_1 = \nu_2$.

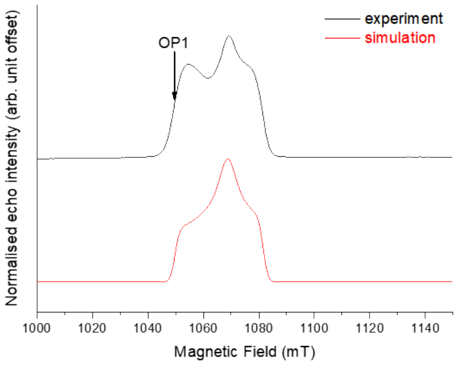

Figure S17. Q-band (34 GHz) echo-detected field-swept EPR spectrum of compound **2** at 3 K. The arrow marks the observer positions where HYSCORE experiments in Figure S18 were performed.

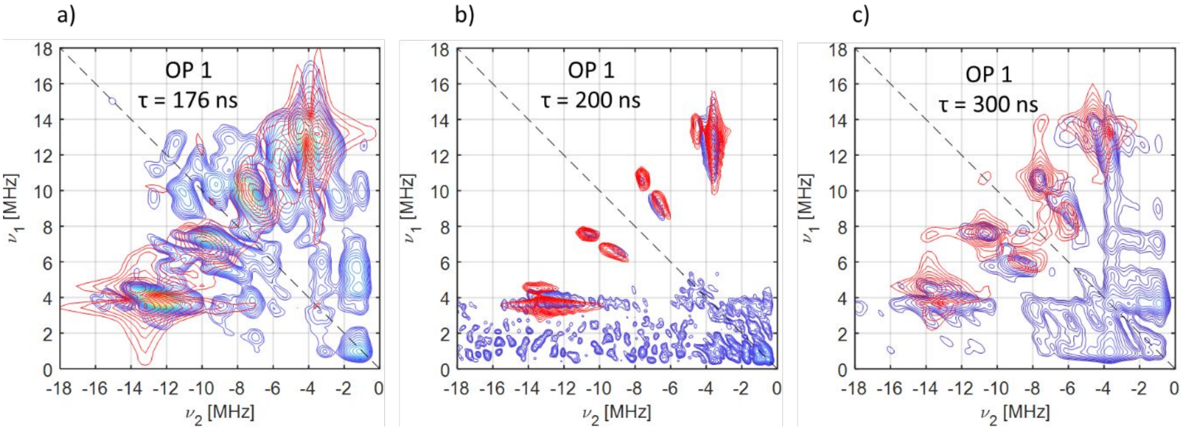

Figure S18. Q-band (34 GHz) experimental (blue) and simulation (red) ^{14}N-HYSCORE spectra of compound **2** at OP1 and 3 K with different interpulse delay. The black dashed line marks the antidiagonal where $-\nu_1 = \nu_2$.

ENDOR

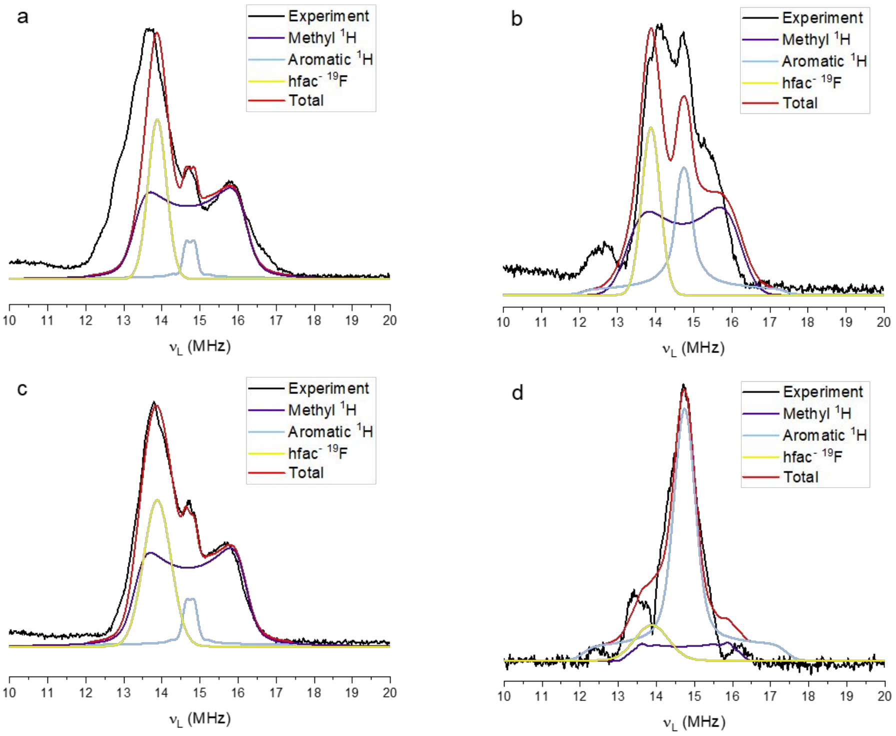

Figure S19. X-band (*ca.* 9.7 GHz) experimental (black) and simulations (see legend) ^{1}H, ^{19}F Mims ENDOR spectra of a 1.2 mmol·L^{-1} solution of **1** at 347.0 mT and 8 K with (a) τ = 118 ns, (b) τ = 136 ns, (c) τ = 200 ns and (d) τ = 300 ns.

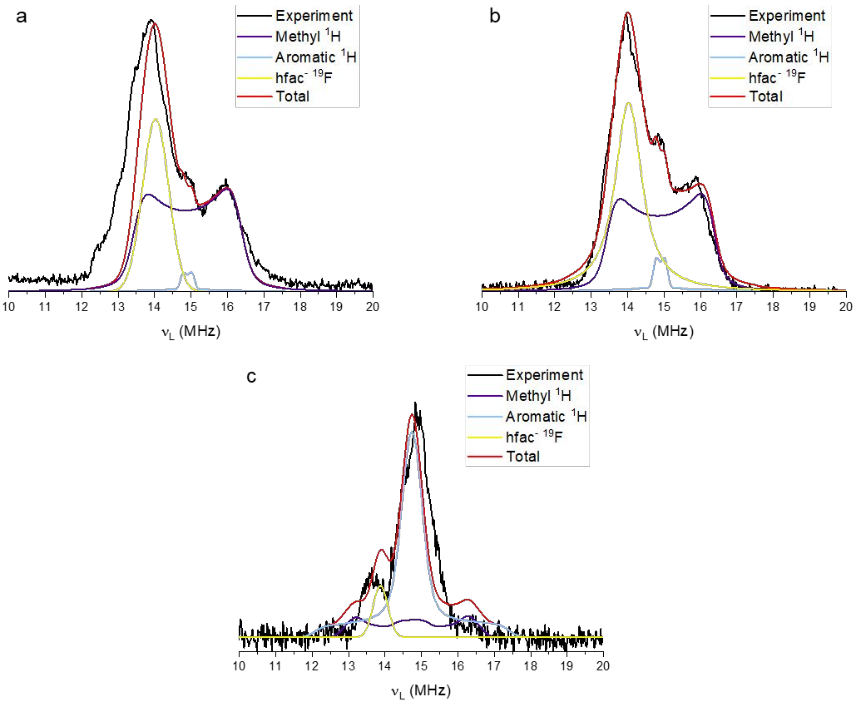

Figure S20. X-band (*ca.* 9.7 GHz) experimental (black) and simulations (see legend) ^{1}H, ^{19}F Mims ENDOR spectra of a 1.2 mmol·L^{-1} solution of **1** at 350.0 mT and 8 K with (a) τ = 118 ns, (b) τ = 300 and (c) τ = 500 ns.

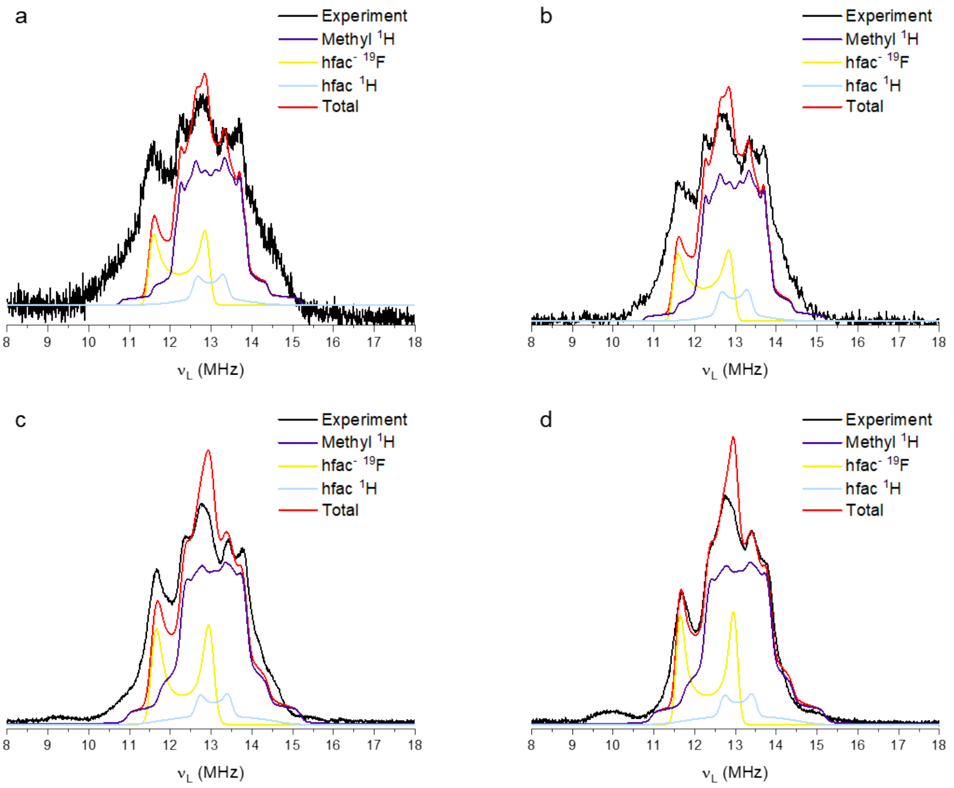

Figure S21. X-band (*ca.* 9.7 GHz) experimental (black) and simulations (see legend) ^{1}H, ^{19}F Mims ENDOR spectra of a 1.2 mmol·L^{-1} solution of **2** at 5.5K, 305.1 mT, (a) τ = 150 ns , (b) τ = 200 ns and at 307.6 mT and (c) τ = 200 ns and (d) τ = 300 ns.

Table S9. Mulliken atomic spin densities

Complex 1, uB3LYP, TZVP

1 Zn 0.004517
2 O -0.000637
3 O 0.217611
4 O 0.000613
5 O 0.001297
6 O 0.000120
7 N 0.253680
8 O 0.000118
9 N 0.299301
10 O 0.409404
11 C 0.021386
12 C -0.186986
13 N -0.000407
14 O 0.000074
15 O -0.000376
16 O 0.000654
17 C -0.015957
18 H 0.000273

19	C	-0.015672
20	C	0.005075
21	F	0.000012
22	C	-0.017338
23	C	-0.005566
24	C	-0.000103
25	C	-0.007858
26	H	0.000338
27	C	0.002997
28	C	-0.000093
29	F	-0.000002
30	F	-0.000001
31	F	0.000009
32	C	-0.000894
33	C	0.000014
34	F	0.000000
35	C	0.000069
36	H	-0.000003
37	F	0.000006
38	C	0.001202
39	H	-0.000264
40	H	-0.000312
41	H	0.000182
42	C	-0.000092
43	F	0.000011
44	C	0.021700
45	H	-0.000347
46	H	0.000782
47	H	-0.001720
48	C	-0.000341
49	H	-0.000654
50	H	-0.000508
51	H	0.000653
52	C	0.000086
53	C	0.000200
54	H	-0.000017
55	F	0.000001
56	C	0.013793
57	H	-0.000503
58	H	0.000711
59	H	-0.000372
60	F	0.000010
61	C	0.000012
62	F	-0.000002
63	F	-0.000002
64	C	0.000111
65	F	0.000001
66	C	-0.000063

67 H 0.000000
68 H 0.000034
69 H 0.000031
Sum of Mulliken atomic spin densities = 1.00000

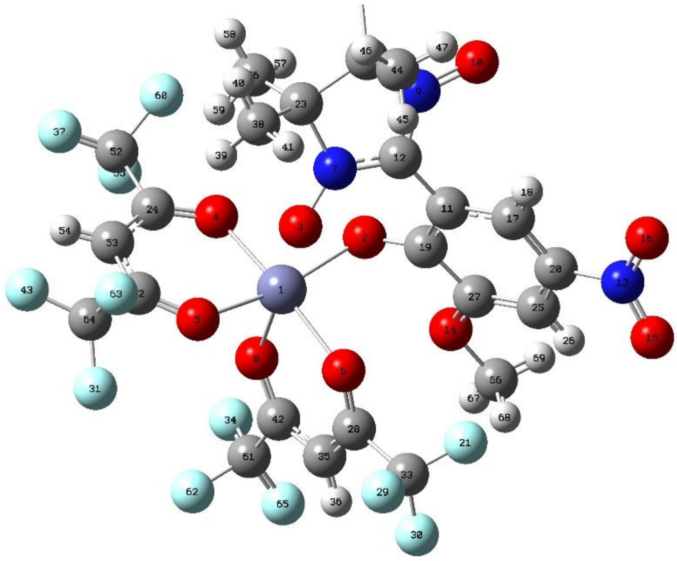

Complex **2**, uB3LYP, TZVP

1 Ni 1.689002
2 O -0.176842
3 O 0.025309
4 O 0.042882
5 O 0.046021
6 O 0.045988
7 O 0.047822
8 N -0.259616
9 C 0.017400
10 C -0.005258
11 C -0.007331
12 C -0.006859
13 C -0.005171
14 C 0.195157
15 C 0.008052
16 C -0.011034
17 C 0.000669
18 C 0.000790
19 C 0.005816
20 C 0.005635
21 C 0.001741
22 C 0.006161
23 C 0.006291
24 N -0.289179
25 C 0.018458
26 C -0.001331
27 C -0.016224

28	C	0.008197
29	O	-0.000443
30	C	0.013054
31	H	-0.000232
32	F	0.000541
33	F	0.000155
34	F	-0.000019
35	F	0.000055
36	F	0.000409
37	F	-0.000099
38	H	-0.000224
39	F	-0.000024
40	F	0.000444
41	F	-0.000110
42	F	0.000166
43	F	0.000083
44	F	0.000406
45	O	-0.401709
46	C	-0.022363
47	C	0.000767
48	H	0.000118
49	H	0.000301
50	H	-0.000113
51	H	0.000476
52	H	-0.000830
53	H	0.000486
54	H	0.000088
55	C	0.005308
56	C	-0.000030
57	H	-0.000537
58	H	0.000292
59	H	-0.000612
60	H	0.001600
61	H	0.000653
62	H	0.000498
63	H	-0.000619
64	N	0.001891
65	H	0.000068
66	H	-0.000012
67	H	0.000037
68	O	0.003610
69	O	0.003922

Sum of Mulliken atomic spin densities = 1.00000

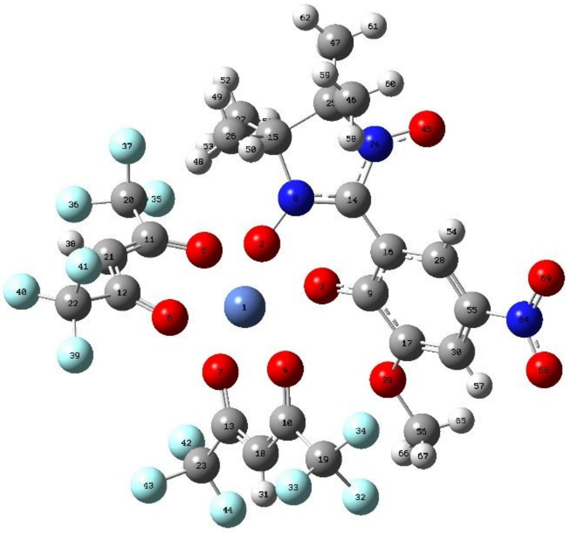

Supplement: Supplementary file 1 [file ic5c05585_si_001.pdf]
